# Mechanically Regulated Nanozymes for Remote Metabolic Reprogramming and Precise Cancer Therapy

**DOI:** 10.1002/anie.8358731

**Published:** 2026-05-26

**Authors:** Fangman Chen, Xiaochun Xie, Hanyao Huang, Ka Hong Wong, Jiying Liu, Hui Fang, Shaowen Wang, Jianfang Cao, Yu Tao, Mingqiang Li, Chao Yang, Wen Sun, Dan Shao, Yunlu Dai

**Affiliations:** ^1^ Cancer Centre and Institute of Translational Medicine, Faculty of Health Sciences University of Macau Macau SAR China; ^2^ MoE Frontiers Science Center for Precision Oncology University of Macau Macau SAR China; ^3^ State Key Laboratory of Oral Diseases and National Clinical Research Center For Oral Diseases and Department of Oral and Maxillofacial Surgery West China Hospital of Stomatology, Sichuan University Chengdu China; ^4^ School of Medicine South China University of Technology Guangzhou China; ^5^ State Key Laboratory of Quality Research in Chinese Medicine, Institute of Chinese Medical Sciences University of Macau Macau SAR China; ^6^ Department of Chemistry University of Michigan Ann Arbor USA; ^7^ State Key Laboratory of Fine Chemicals, School of Chemical Engineering Dalian University of Technology Dalian China; ^8^ Laboratory of Biomaterials and Translational Medicine, Department of Ultrasound, Center For Nanomedicine The Third Affiliated Hospital Sun Yat‐sen University Guangzhou China; ^9^ Department of Orthopedics, Center For Orthopedic Surgery The Third Affiliated Hospital of Southern Medical University Guangzhou China

**Keywords:** cascade catalysis, mechanical force, mechanical regulation, nanozyme, tumor therapy

## Abstract

Mechanoenzymes, featuring catalytic activity controlled by mechanical stimuli, play key roles in maintaining metabolic order and cellular homeostasis. However, artificial nanozymes with strict spatiotemporal regulation are still rare, limiting their effectiveness in complex biological environments. Here, we introduce a mechanically regulated nanozyme (MRNZ) by integrating mechano‐responsive ferrocene (Fc) units into a flexible framework. Similar to natural enzymatic activation, acoustic shear forces cause sub‐nanostructural transformations of Fc units, leading to decreased electron density and reduced steric hindrance at Fe active sites, reinforcing metabolic peroxidase (POD)‐like activity. This mechanical activation enables precise modulation of metabolic reprogramming by controlled generation of low‐dose hydroxyl radicals (•OH) as second messengers, improving stem cells resilience to oxidative stress for safer and more effective therapeutic interventions. Using this mechanically regulated method, we encapsulated glucose oxidase (GOx) inside hollow MRNZ to create a multienzyme regulated nanoreactor (MRNZ@GOx) that orchestrates a cascade GOx‐POD reaction under ultrasound stimulation. Such a cascade reactive oxygen species generation in tumor microenvironments potentiates chemodynamic therapy combined with immune activation. Our work introduces a mechanically responsive strategy for regulating nanozyme activity, expanding the horizons of next‐generation remote and smart catalytic technologies for precise disease treatments.

## Graphical abstract

Inspired by mechanoenzymes, a ferrocene‐based mechanically regulated nanozyme converts acoustic shear force into enhanced peroxidase‐like activity by decreasing electron density and alleviating steric hindrance at the Fe active sites, thereby rewiring redox homeostasis. Leveraging this remote regulatory mechanism, a multienzyme‐regulated nanoreactor is constructed to amplify chemodynamic effects, broadening the scope of catalytic technologies for precise cancer therapy.

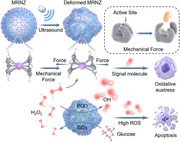

## 1 Introduction

Natural enzymes exhibit impressive catalytic efficiency and substrate selectivity, making their broad utility in biomedicine [1, 2, 3]. However, their use in clinics and industry are limited by problems like poor storage stability, weak chemical durability, possible immunogenicity, and high production cost [4]. To overcome these problems, artificial nanozymes, engineered nanomaterials that mimic enzymatic catalytic activities, have become promising alternatives [5, 6, 7, 8]. Despite their advances, most artificial nanozymes lack the precise control characteristic of natural enzymes. This makes them less useful in changing and complicated biological environments. Efforts to improve nanozyme performance have mostly focused on stimuli‐responsive regulation [9, 10]. Among them, photoresponsive systems allow light‐controlled modulation of activity [11, 12]. But ultraviolet or visible light regulation suffers from limited tissue penetration, potential genetic damage, and indirect structure‐mediated effects on active sites [13, 14]. These challenges show needing remote, adjustable strategies that can accurately and reversibly control nanozyme activity in living systems [15].

In this context, mechanical force, a fundamental biophysical regulator, offers a compelling alternative [16, 17, 18]. Mechanical forces are common modulators of enzymatic activity in nature, often acting through mechanical regulation mechanisms that reshape active‐site geometry [19]. For instance, shear stress in blood flow activates coagulation factors during hemostasis, while cellular traction enhances the proteolytic output of matrix metalloproteinases [20, 21, 22, 23, 24, 25]. Ultrasound (US) can deliver such forces non‐invasively, with superior penetration depth compared to light and greater specificity and safety than bulk heating [26]. Previous studies have demonstrated that acoustic shear forces can influence the conformational dynamics of engineered enzymes, suggesting a feasible route toward externally controlled catalysis via mechanochemically induced structural rearrangements [27, 28]. This approach could endow artificial nanozymes with enzyme‐like mechanical regulation behavior, enabling their deployment in complex biological systems such as metabolic reprogramming or therapeutic interventions.

Mechanophores, molecular motifs that undergo specific structural transformations under mechanical stress, offer a design pathway toward such control [29, 30]. When incorporated into polymeric backbones, mechanophores can activate catalytic sites or trigger chemical reactions in response to external forces [31]. Guided by this principle, we sought to introduce mechanical regulation into artificial nanozymes by embedding mechano‐responsive ferrocene (Fc) units within a flexible nanocapsule framework. We hypothesized that ultrasound‐induced mechanical stress could trigger sub‐nanometer conformational transformations, thereby modulating local electron density and steric configuration at Fe catalytic centers with high precision.

**SCHEME 1:**
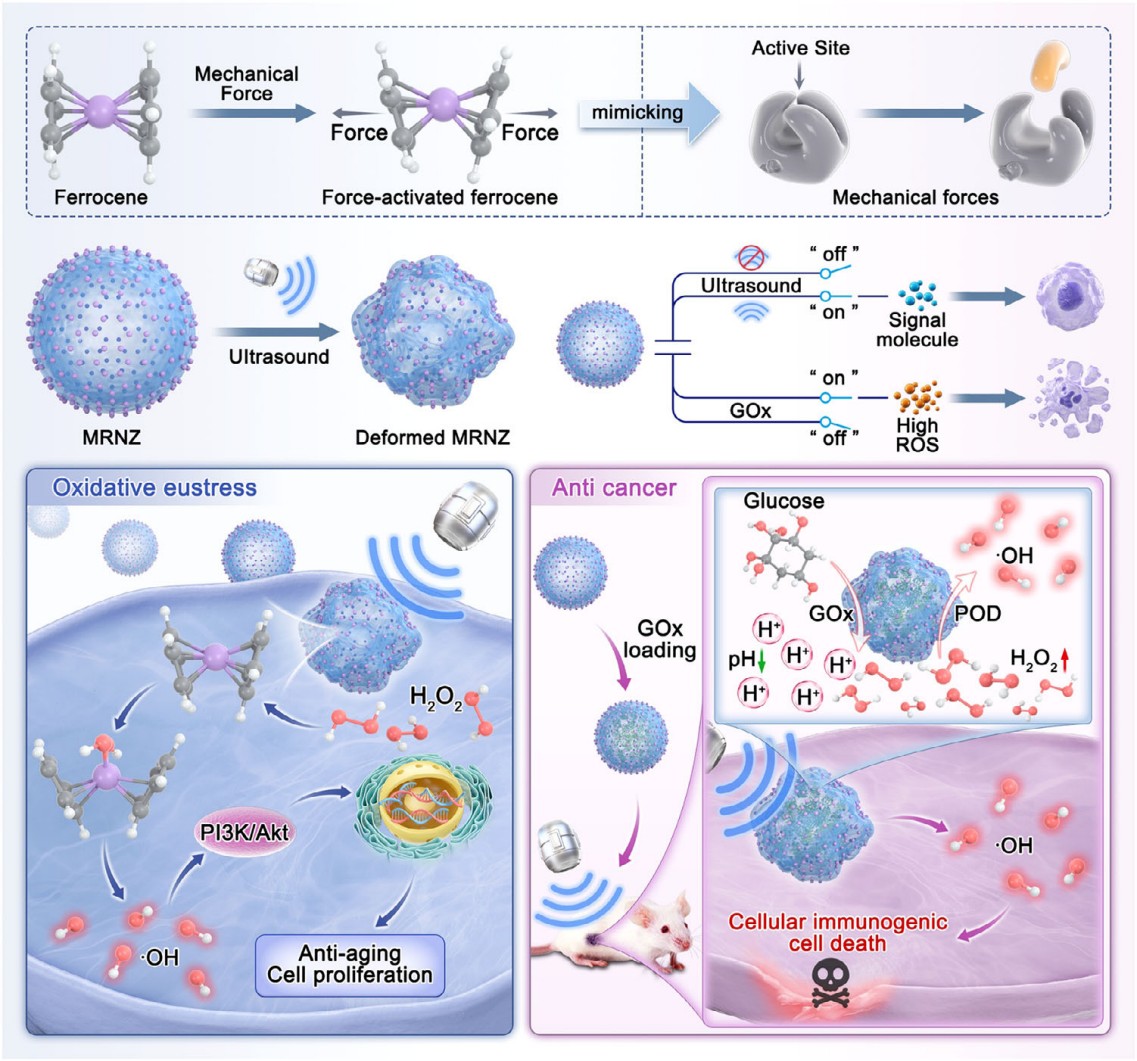
Schematic illustration of the mechanically regulated nanozyme (MRNZ) designed to mimicking natural mechanoenzymes. Mechanical regulation activates intrinsic catalytic sites within the MRNZ to produce •OH on demand upon mechanochemical stimulation. Low‐dose •OH as second messengers permit targeted modulation of cellular metabolic pathways, simultaneously, an integrated GOx‐POD cascade amplifies ROS generation for chemodynamic therapy.

To test this hypothesis, we developed a mechanically regulated nanozyme (MRNZ) by integrating Fc mechanophores into a deformable nanocapsule backbone (Scheme 1). This design provides strong mechano‐responsivity and abundant reactive sites for efficient catalysis. When exposed to US, acoustic shear forces stretch the Fc units within MRNZ, causing sub‐nanostructural transformations that reduce electron density and steric hindrance at the Fe active sites. This process, analogous to natural enzyme mechano‐regulation, enhances electron transfer efficiency and optimizes substrate adsorption and product desorption energetics, thereby enabling on/off switching of peroxidase (POD)‐like activity with stringent spatiotemporal precision. We further demonstrate that this mechanochemical process allows precise regulation of intracellular reactive oxygen species (ROS). It enables controlled generation of low‐dose hydroxyl radicals (•OH) as second messengers under physiological conditions, enhancing stem cell resilience to oxidative stress. By using this mechanical regulation, we further packed glucose oxidase (GOx) inside the hollow MRNZ to construct a multi‐enzyme regulated nanoreactor (MRNZ@GOx). Under ultrasound irradiation, MRNZ@GOx orchestrates a cascade GOx‐POD reaction that amplifies the production of ROS in tumor microenvironments (TMEs). This cascade induces immunogenic cell death (ICD), increases PD‐L1 checkpoint blockade, and achieves potent chemodynamic therapy combined with immune activation. Collectively, this work establishes a mechanically responsive method for regulating nanozyme activity, offering a versatile platform for precise metabolic modulation and intelligent therapeutic interventions in complex biological systems.

## 2 Results and Discussion

**FIGURE 1:**
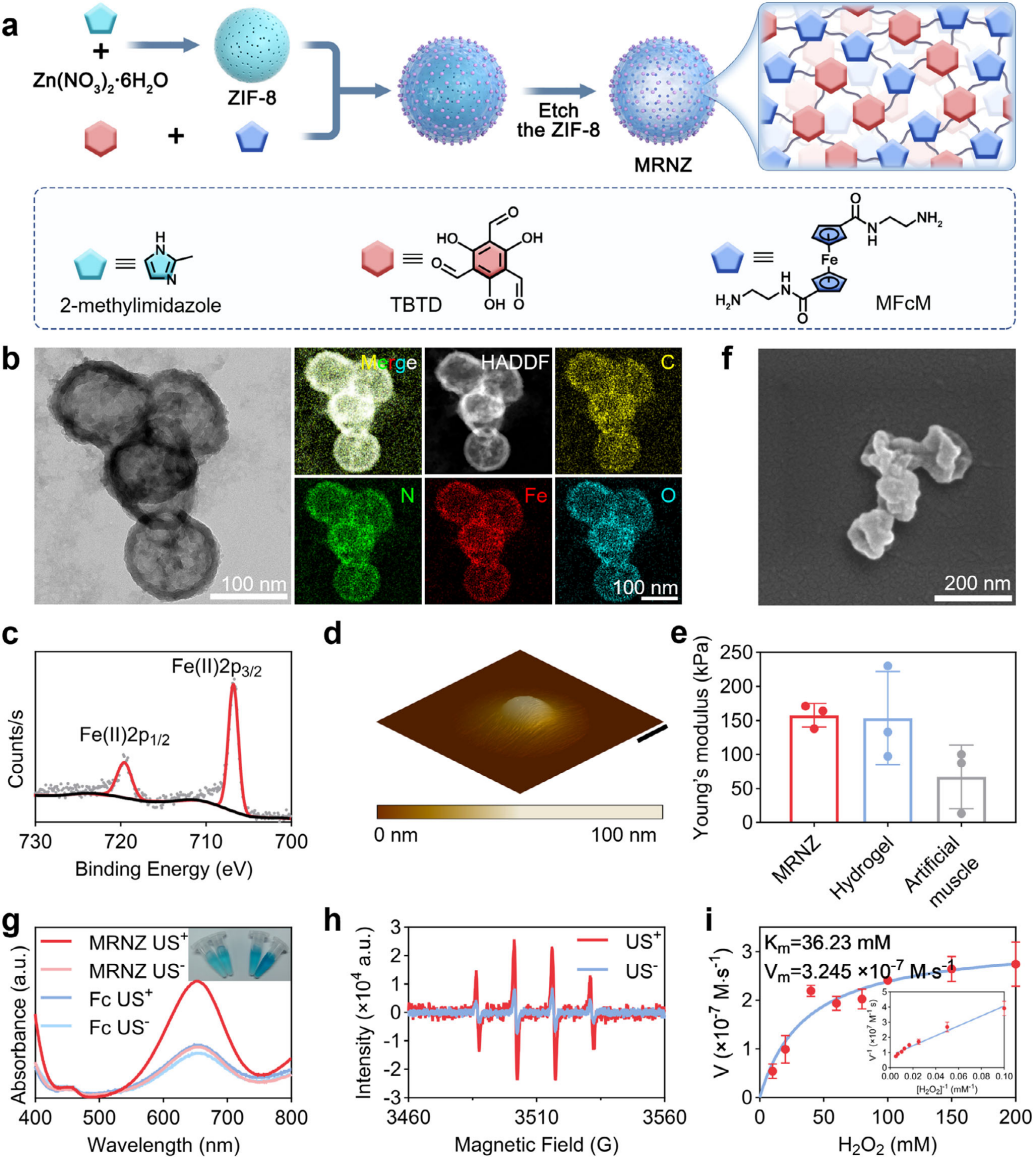
(a) Schematic illustration of the synthesis of MRNZ. (b) TEM image of MRNZ and the HAADF‐STEM and EDX‐mapping. (c) Fe 2p XPS spectra of MRNZ and their corresponding fitting curves. (d) AFM height profiles of MRNZ. Scale bars, 100 nm. (e) Young's modulus profiles of previously reported material [32, 33, 34]. (f) SEM image of MRNZ after US treatment. (g) UV absorption spectra of TMB with different treatment. (h) ESR spectra of •OH from MRNZ without and with US treatment irradiation. (i) The POD‐like activity of MRNZ with US. Typical Michaelis‐Menten curves with various concentrations of H_2_O_2_ in NaOAc‐HOAc buffer (100 mM, pH 6.5), illustration: the Lineweaver‐Burk fitting (double reciprocal) of Michaelis‐Menten fitting curve activity. Data are mean ± SD (*n* = 3).

We synthesized a mechano‑responsive Fc monomer and confirmed its structure using ^1^H‐NMR, mass spectrometry and Fourier transform infrared (FT‐IR) spectroscopy (Figure S1). The Fc monomer was subsequently conjugated with 9H‐fluoren‐9‐ylmethyl N‐(2‐aminoethyl) carbamate (EDA) to yield an Fc‐containing precursor. The MRNZ was fabricated by covalent cross‐linking of this Fc‐containing precursor on the surface of a zeolitic imidazolate framework‐8 (ZIF‐8) sacrificial template (Figures 1a and S2a–d). The resulting product displayed uniform spherical morphology with a hydrodynamic diameter of 125 ± 10 nm (Figure S2e) and a ζ potential of −23.50 ± 5 mV (Figure S2f). Transmission electron microscopy (TEM) revealed a hollow architecture with a shell thickness of 16 ± 2 nm (Figure 1b). Nitrogen adsorption‐desorption isotherms displayed a type‐IV profile with hysteresis, and Brunauer‐Emmett‐Teller (BET) analysis indicated mesopores of 4 ± 2 nm, favorable for efficient substrate diffusion (Figure S2g). Elemental analysis by energy‑dispersive x‑ray spectroscopy (EDS) detected C, N, Fe and O and, along with the hollow structure, indicated complete removal of the ZIF‑8 core (Figure S2h). Elemental mapping further showed uniform distribution of the Fc units throughout MRNZ (Figures 1b and S2i). FT‑IR spectra exhibited a new absorption band at 1622 cm^−1^ (C = N stretching), while the C = O (1697 cm^−1^) and N‐H (3300–3500 cm^−1^) bands disappeared, confirming the formation of imine linkages in the capsule backbone (Figure S2j). X‑ray photoelectron spectroscopy (XPS) showed Fe 2p3/2 and Fe 2p1/2 peaks at 710.28 and 723.88 eV, respectively, verifying successful incorporation of Fc while retaining its mechanophore identity (Figure 1c). Inductively coupled plasma mass spectrometry (ICP‑MS) measured an Fe content of 15 ± 3wt%, suggesting a high density of reactive sites for mechano‐responsivity and catalysis. In addition, atomic force microscopy (AFM) gave an average particle height (apparent thickness) of 125 ± 5 nm (Figure 1d). AFM nanoindentation analyzed using the Hertz model, yielded a Young's modulus of 157.67 kPa that comparable to soft hydrogels but substantially lower than rigid nanoparticles [32, 33, 34], underlining the capsule's mechanical flexibility (Figures 1e and S3). After US exposure, scanning electron microscope (SEM) showed visible capsule deformation, matching the mechano‐responsive design (Figure 1f).

Mechanical stretching of MRNZ caused sub‐nanostructural rearrangements. This made its catalytic activity adjustable when exposed to acoustic shear forces under US irradiation. The oxidative activity of MRNZ greatly increased upon US stimulation, far exceeding that of MRNZ alone, a physical mixture of nanocapsule particles and free Fc (NP + Fc), a nanocapsule system with the same Fc loading in which Fc is covalently linked through only one cyclopentadienyl (Cp) ligand (NP/Fc) or US treatment alone (Figures 1g and S4a–c). The characteristic oxidation of 3,3′,5,5′‐tetramethylbenzidine (TMB) intensified with prolonged US exposure, and the catalytic activity could be reversibly modulated through intermittent US irradiation (on/off cycles), demonstrating precise, reversible catalytic regulation (Figure S4d). Interestingly, US irradiation caused minimal temperature elevation, indicating that the enhanced catalytic oxidation activity originated primarily from acoustic mechanical force causing changes in sub‐nanostructural transformations, not from heat (Figure S4e). Electron spin resonance (ESR) tests with specific trapping agents confirmed that •OH were the dominant ROS, confirming the mechano‐enhanced POD‐like activity of MRNZ (Figure 1h). Steady‐state kinetic assays using H_2_O_2_ as the substrate further revealed that MRNZ under US exhibited a lower Michaelis constant (*K_m_*) and a higher maximum velocity (*V*_m_) than MRNZ alone, indicating both enhanced substrate affinity and improved catalytic turnover. Specifically, US stimulation increased affinity and catalytic efficiency by factors of 2.70 and 1.71, respectively (Figure 1i and S4f). Overall, these results establish that acoustic shear forces enable reversible switching of POD‐like activity, enabling on‐demand •OH generation with high spatiotemporal precision.

**FIGURE 2:**
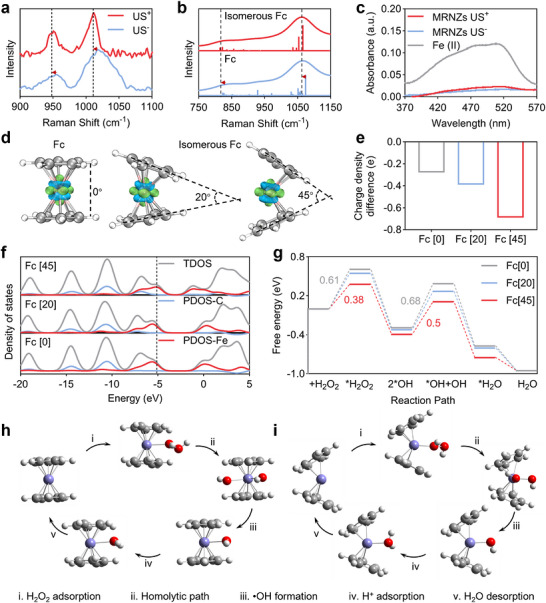
(a) The in situ Raman spectrum of MRNZ before and during treatment of US. (b) The simulated Raman spectrum of Fc and isomerous Fc with full width at half maximum (FWHM) of 100 cm^−1^. (c) UV absorption spectra of the [Fe(phenanthroline)_3_]^2+^ complex. (d) Illustration of optimization of different Fc structure models with respective dihedral angle of Cp‐Fe‐Cp (*α* = 0°, 20°, and 45°). (e) Charge density difference of Fc structure with different angle transition. (f) DOS of Fc with different angle. (g) Corresponding free energy diagram for Fenton reaction of Fc with different conformational transition. (h) Proposed catalytic mechanism for Fenton reaction of Fc (*α* = 0°). (i) Proposed catalytic mechanism for Fenton reaction of Fc (*α* = 45°).

Covalent incorporation of Fc mechanophores within the MRNZ framework ensures efficient transmission of mechanical strain to the force‐responsive centers, driving sub‐nanometer rearrangements. We reasoned that acoustic shear force would peel the Cp ligands and widen the Cp‐Fe‐Cp dihedral angle (*α*), as steric constraints within the nanocapsule restrict ligand rotation. Raman spectroscopy supported this mechanism, the Cp‐Fe‐Cp breathing and vibrational modes at 954.87 and 1017.34 cm^−^^1^ red‐shifted to 950.69 and 1011.12 cm^−^^1^ under US irradiation, whereas the C = O stretch at 1422.71 cm^−^^1^ remained unchanged (Figure 2a). Theoretical Raman simulations displayed consistent red‐shifts, corroborating mechanical deformation of Fc (Figures 2b and S5). Iron leaching was ruled out, as the 1,10‐phenanthroline adduct showed no detectable Fe^2+^ chromophore at 520 nm after US treatment (Figure 2c). Together, these data indicate that mechanical force stretches and twists the Fc unit within the MRNZ, peeling Cp ligands to increase the dihedral angle (*α*), and expose Fe active centers without Fe^2+^ dissociation. Density functional theory (DFT) calculations further resolved the structural evolution of the Cp‐Fe‐Cp motif at the defined dihedral angle *α* (*α* = 0°, 20°, and 45°) along the Cp‐Fe‐Cp axis (Supporting Information, details of computational methods therein, Figure 2d). Charge‐density difference maps for increasing *α* (0°, 20°, 45°) revealed weakened Fe‐Cp interactions and reduced electron density around Fe (Figure 2e). Corresponding density‐of‐states (DOS) analyses showed a more continuous electronic distribution near the Fermi level and new hybridized states, indicating enhanced charge‐transfer capacity at stretched sites (Figures 2f and S6a). These results indicated that mechanical stretching of MRNZ trigger sub‐nanostructural transformations that modulate both steric accessibility and electron density at the Fc active sites. To correlate these sub‐nanostructural changes with catalysis, we modeled the H_2_O_2_ decomposition pathway underlying the POD‐like activity of MRNZ. Calculated Gibbs free‐energy profiles and key intermediates along the optimized H_2_O_2_→•OH route showed that substrate adsorption at Fe centers became increasingly favorable with larger *α* values, reflected by lower energy barriers and shorter Fe‐O bond lengths (Figures 2g–i and S6b–e). Subsequent O‐O bond cleavage to form *OH intermediates proceeded more readily at larger dihedral angles *α* (Figure S6d). The rate‐limiting desorption of *OH experiences the largest barrier reduction under mechanical strain (Figure 2g). Overall, these spectroscopic and computational results demonstrated that mechanical stretching of Fc mechanophores induced enzyme‐like sub‐nanometer rearrangements. This mechanical regulation improved electron transfer, optimized substrate binding, and enhanced product release, enabling reversible and precise control over nanozyme activity. Such behavior closely mirrors natural enzymatic regulation and underlines a promising strategy for bridging artificial and biological catalysis.

**FIGURE 3:**
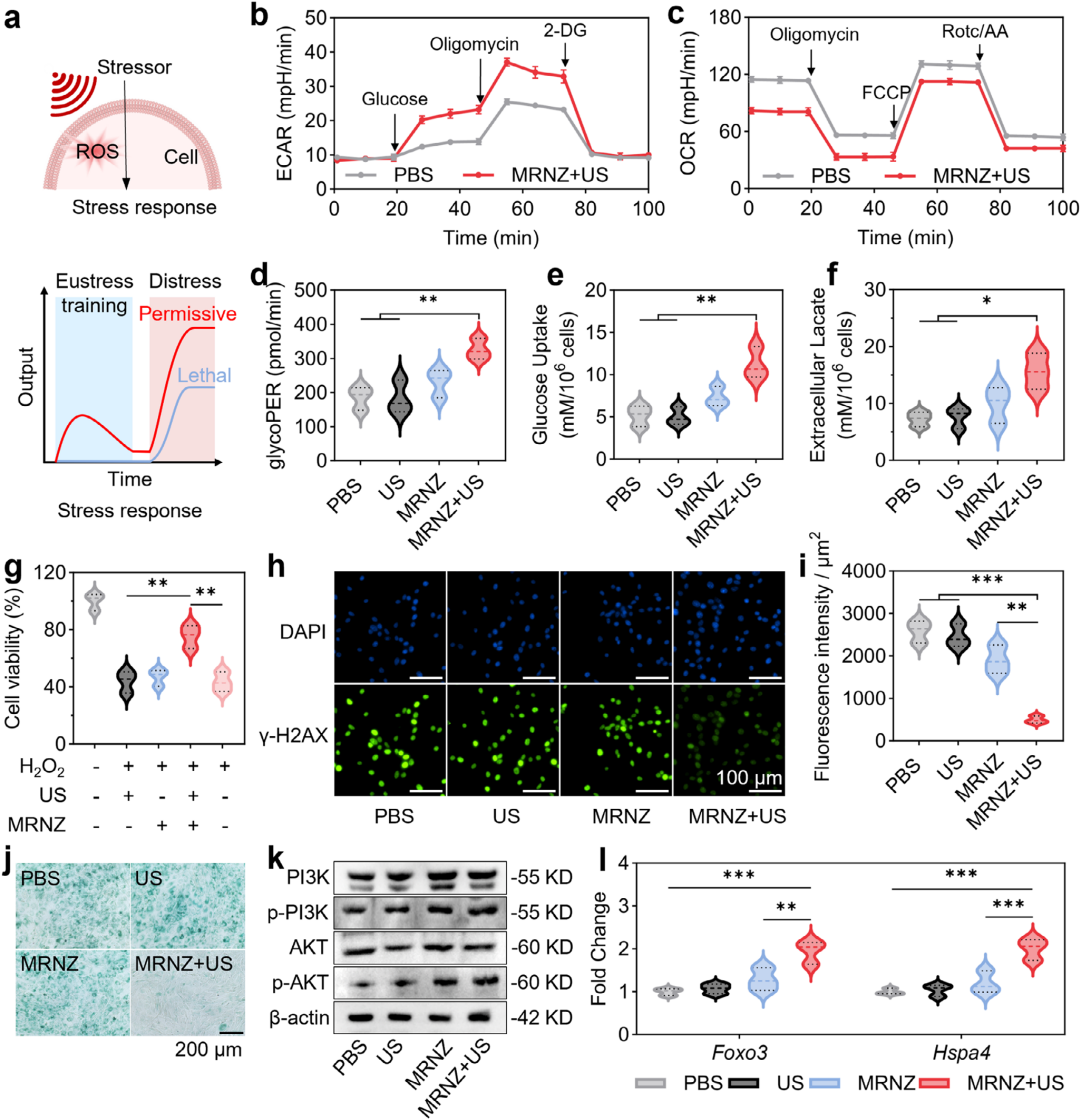
(a) Oxidative eustress training increases cell stress adaptation. (b) MSCs were treated with different treatment and glucose (100 mM), oligomycin (10 µM), and 2‐DG (500 mM) were successively added to measure ECAR. (c) MSCs were treated with different treatments: oligomycin (1.5 µM), FCCP (1 µM) and rotenone/antimycin A (0.5 µM) were successively added to measure OCR. (d) Basal glycolysis of MSCs with different treatments. (e) Glucose uptake of MSCs with different treatments. (f) Extracellular lactate of MSCs with different treatments. (g) MSCs viability, (h, i) immunofluorescence of γ‐H2AX, and (j) senescence‐associated beta‐galactosidase (SA‐beta‐gal) staining following preconditioning with MRNZ and exposure to 200 µM H_2_O_2_ for 1 h. (k) Western blots of PI3K/AKT pathway members. β‐Actin was used as the internal reference. (l) Expression fold change of *Foxo3* and *Hspa4* in MSCs with different treatment. Data are mean ± SD (*n* = 3); *p < 0.05, **p < 0.01, ***p < 0.001 were assessed via one‐way analysis of variance (ANOVA) or two‐way ANOVA with Tukey's multiple comparison tests.

The •OH are often viewed as highly reactive and destructive species. However, when generated in a spatially and temporally controlled manner, moderate •OH levels can act as secondary messengers. These reactive intermediates covalently modify key proteins in signaling pathways, tuning enzymatic activity and transcriptional programs involved in metabolic regulation and stress adaptation [35, 36, 37]. This form of controlled oxidative signaling, known as oxidative eustress, enhances cellular resilience to stress and helps maintain homeostasis (Figure 3a). MRNZ showed excellent colloidal stability (Figure S7a). To investigate whether mechanically regulated catalysis by MRNZ modulates cellular metabolism, we assessed extracellular acidification rate (ECAR) and oxygen consumption rate (OCR) as indicators of glycolytic and oxidative phosphorylation (OXPHOS) activities, respectively. Seahorse flux tests showed that MRNZ treated mesenchymal stem cells (MSCs) quickly increased ECAR within 5 min of US irradiation, along with a decrease in OCR (Figures 3b,c and S7b,c). This metabolic shift reflects a pronounced bias toward glycolysis, characterized by the increased basal glycolysis and glycolytic capacity (Figures 3d and S7d), along with a substantial reduction in glycolytic reserve. Consistent with these findings, intracellular metabolite profiling showed increased glucose uptake and lactate secretion in MRNZ treated MSCs upon US irradiation (Figure 3e,f). To check if this glycolytic upregulation was required for cell survival, MSCs were treated with the glycolytic inhibitor 3‐bromopyruvic acid (3‐BrPA) for 48 h. Even with enough oxygen, MRNZ + US treated MSCs exhibited a strong dependence on glycolysis for survival (Figure S8a). In contrast, treatment with US or MRNZ alone had negligible effects on metabolism, confirming that programmable acoustic catalytic process, not heat, drove the adaptive glycolytic reprogramming in MSCs.

We next explored the potential of this adaptive glycolytic reprogramming by programmable acoustic catalytic process to enhance the adaptive capacity of MSCs against oxidative stress. MSCs showed no detectable cytotoxicity when exposed to varying concentrations of MRNZ under 5 min of US irradiation, confirming its safety of the system (Figure S8b,c). Importantly, pretreatment with MRNZ combined with US irradiation greatly reduced H_2_O_2_‐induced cytotoxicity and increased cell viability compared to untreated controls (Figure 3g). Further analyses revealed that this pretreatment substantially mitigated nuclear DNA damage (Figure 3h,i), cellular senescence (Figures 3j and S9a), and senescence‐associated stress response genes (Figure S9b–f). These results indicate a pronounced enhancement of oxidative stress tolerance in MRNZ + US preconditioned MSCs. Mechanistically, western blot (WB) analysis confirmed the activation of the PI3K‐Akt signaling pathway in MRNZ + US pretreated MSCs (Figures 3k and S10), a key axis known to support cell survival and metabolic changes [35]. In addition, quantitative reverse transcription PCR showed much higher expression levels of downstream effectors *Foxo3* and *Hspa4* (Figure 3l), both closely tied to handling oxidative stress. *Foxo3* was known to activate the transcription of genes encoding antioxidant enzymes that removed ROS, while *Hspa4* prevented the aggregation of misfolded proteins and promoted the repair or degradation of damaged proteins [38]. Notably, the protective effects of MRNZ + US pretreatment were abolished upon inhibition of the PI3K‐Akt pathway with HY‐15727, confirming that this signaling axis was essential for the acoustic catalysis‐induced oxidative eustress response (Figure S9g). Taken together, these results demonstrated that mechanically regulated catalysis via MRNZ acted as a programmable acoustic modulator that enhanced oxidative stress adaptation by driving metabolic reprogramming. This non‐destructive strategy enabled controlled activation of protective signaling pathways and may help prevent MSC transplantation from oxidative apoptosis and premature senescence.

**FIGURE 4:**
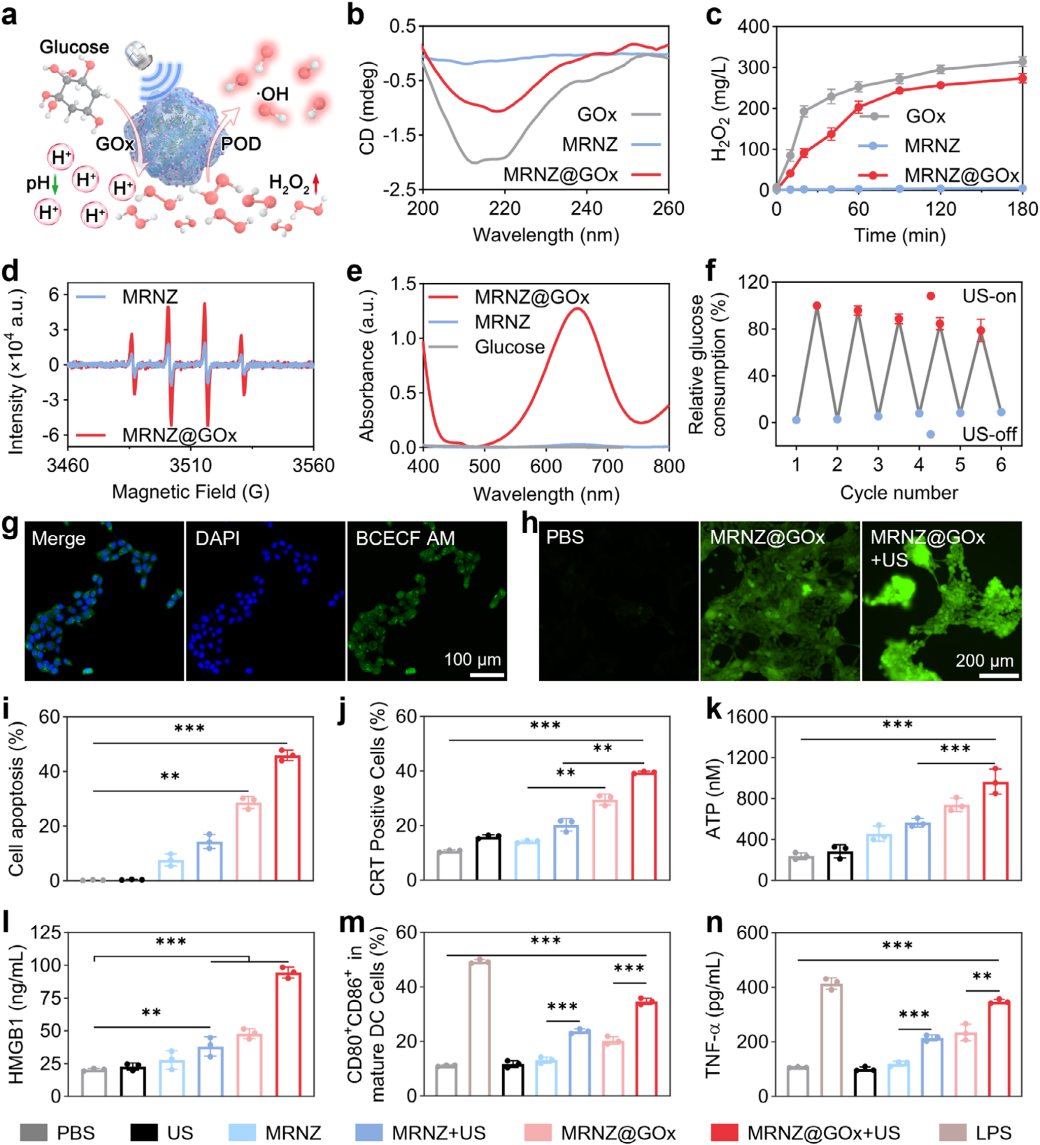
(a) Catalytic cascade of MRNZ@GOx combined GOx enzyme and POD‐like activities with US functioning as an on‐off switch. (b) CD spectra of GOx and MRNZ@GOx. (c) Time‐dependent H_2_O_2_ production (*n* = 3). (d) ESR spectra of •OH generation, and (e) UV absorption spectra of TMB in glucose solution under US irradiation. (f) “off‐on” switching of catalytic activities of MRNZ@GOx (*n* = 3). (g) Confocal fluorescent microscopy images of BCECF AM in 4T1 treated with MRNZ@GOx + US. (h) Fluorescent microscopy images of H2DCF‐DA (green) in treated 4T1 cells. (i) Apoptosis, (j) CRT exposure, (k) ATP levels, and (l) HMGB1 release in 4T1 cells under different treatments. (m) Percentage of CD80^+^CD86^+^ expression and (n) TNF‐α secretion levels in BMDCs after coincubation with treated 4T1 cells. Data are mean ± SD (*n* = 3); *p < 0.05, **p < 0.01, ***p < 0.001 were assessed via one‐way ANOVA with Tukey's multiple comparison tests.

Excessive •OH production can inflict severe oxidative damage to cellular components. To take advantage of therapeutic purposes, we engineered a MRNZ@GOx by encapsulating GOx within the hollow MRNZ framework [39]. This design enables cascade catalytic amplification of •OH generation within the TME (Figure 4a). Circular dichroism (CD) spectra confirmed successful GOx encapsulation, with minimal perturbation to its secondary structure (Figures 4b and S11a). The hydrodynamic diameter and ζ‐potential of MRNZ@GOx closely matched those of pristine MRNZ (Figure S2e,f), suggesting that GOx was confined within the internal cavities rather than adsorbed on the surface. The GOx loading content reached 23.67 wt%, and its retained catalytic activity was 86.8% of that of the free enzyme (Figure 4c), indicating minimal conformational or functional compromise during incorporation. Upon glucose addition, MRNZ@GOx catalyzed the oxidation of glucose to gluconic acid and H_2_O_2_ in a concentration‐ and time‐dependent manner (Figure S11b–d). The continuous generation of H_2_O_2_ served as an in situ substrate supply for the subsequent POD‐like catalytic step, leading to sustained •OH production (Figure 4c,d). In contrast, negligible colorimetric response toward TMB was detected in the absence of either glucose or GOx (Figures 4e and S11e), confirming that glucose oxidation‐derived H_2_O_2_ is essential for cascade‐driven •OH generation. In the presence of glucose, GOx catalysis initiated the primary oxidation step, while US irradiation amplified both enzyme activities simultaneously. The enhancement in GOx activity might be attributed to mechanically induced deformation of the nanocapsule that increased nanochannel permeability, together with acoustic cavitation effects that accelerated small‐molecule diffusion. The cascade catalytic activities of MRNZ@GOx were found to be mechanically tunable through a US‐triggered “off‐on” switching mechanism (Figure 4f). Upon US exposure, the POD‐like activity of MRNZ@GOx was enhanced by approximately 5.56‐fold compared to that observed without US exposure (Figure S11f). Collectively, MRNZ@GOx acted as a programmable cascade catalytic system, offering precise and intelligent catalytic control for therapeutic applications. The coupled GOx and POD‐like activities can therefore be coordinated with high spatiotemporal precision, closely resembling mechanical regulation in natural enzymatic systems.

Encouragingly, MRNZ@GOx showed excellent colloidal stability in a range of physiological media, including water, phosphate‐buffered saline (PBS), fetal bovine serum (FBS), and complete cell culture medium, with no detectable aggregation over 72 h (Figure S11g). To investigate cellular internalization, 4T1 cells were incubated with Cy5.5‐labeled MRNZ@GOx, followed by confocal laser scanning microscopy (CLSM) imaging. Pronounced co‐localization between Cy5.5 fluorescence and lysosomal markers indicated efficient cellular uptake through the endocytic pathway (Figure S11h). The intracellular catalytic activity of MRNZ@GOx was then investigated. The GOx‐mediated glucose oxidation within cells was assessed by monitoring intracellular acidification using pH probe (BCECF AM), a pH‐sensitive fluorescent probe [40]. CLSM images revealed a pronounced drop in intracellular pH to approximately 4.8 in MRNZ@GOx‐treated cells (Figures 4g and S12), demonstrating strong conversion of glucose to gluconic acid and H_2_O_2_. The in situ generation of H_2_O_2_ supplied the substrate for the subsequent POD‐like catalytic step, enabling a self‐sustaining cascade. Intracellular ROS levels were further evaluated using 2′,7′‐dichlorofluorescein diacetate (DCFH‐DA) as a fluorescence probe. As shown in Figures 4h and S13, 4T1 cells treated with MRNZ@GOx under US irradiation displayed a pronounced increase in DCF fluorescence compared to cells treated with MRNZ@GOx, MRNZ, MRNZ + US, or US alone. This indicated much higher intracellular ROS production under mechano‐regulated conditions. These findings demonstrated that shear force‐induced sub‐nanostructural transformations within MRNZ@GOx synergistically coordinate GOx and POD‐like catalytic activities, enabling mechanically regulated control of enzyme‐mimetic catalysis inside living cells.

The of MRNZ@GOx was assessed by the cell counting kit‐8 (CCK‐8) assay. MRNZ@GOx alone exhibited negligible cytotoxicity toward 4T1 cells, even at concentrations up to 100 µg/mL (Figure S14a), confirming its excellent biocompatibility. In contrast, US irradiation markedly enhanced the cytotoxicity of MRNZ@GOx in a dose‐dependent manner, reducing cell viability to 50% at 79.43 µg/mL. Notably, the addition of the ROS scavenger, L‐ascorbic acid (AA), partially rescued cell viability, demonstrating that the observed cytotoxicity primarily raised from catalysis‐induced oxidative stress (Figure S14b). MRNZ@GOx + US treatment produced substantially greater cytotoxicity than MRNZ + US, underlining the importance of in situ H_2_O_2_ self‐supply through the GOx oxidation cascade. Flow cytometric analysis also confirmed that MRNZ@GOx under US irradiation induced pronounced apoptosis, with apoptotic cell populations reaching approximately 46.90% (Figures 4i and S15). Collectively, these results demonstrated that ultrasound‐enhanced cascade catalysis enabled potent, controllable chemodynamic tumor cell killing through the synergistic action of GOx and POD‐like catalytic pathways.

Beyond direct cytotoxicity, ROS‐mediated apoptosis is well known to initiate ICD. This process involves the exposure or release of damage‐associated molecular patterns (DAMPs), including calreticulin (CRT), adenosine triphosphate (ATP), and high‐mobility group box 1 (HMGB1). These signals play a central role in activating antitumor immune responses. Treatment with MRNZ@GOx + US obviously increased CRT exposure on the surface of 4T1 cells compared with PBS controls, as shown by both immunofluorescence microscopy and flow cytometry (Figures 4j and S16). Similarly, extracellular ATP and HMGB1 levels rose considerably after MRNZ@GOx + US treatment (Figure 4k,l), validating the induction of ICD. To evaluate the immunostimulatory effect, bone marrow‐derived dendritic cells (BMDCs) were co‐cultured with MRNZ@GOx + US‐treated 4T1 cells (Figures 4m and S17a,b). A pronounced upregulation of the costimulatory molecules CD80 and CD86 indicated effective dendritic cell (DC) maturation in response to DAMP release. In parallel, the co‐culture medium showed higher levels of proinflammatory cytokines tumor necrosis factor‐α (TNF‐α) and interleukin‐6 (IL‐6), accompanied by a decrease in the anti‐inflammatory cytokine interleukin‐10 (IL‐10) (Figures 4n and S17c,d). These results established MRNZ@GOx as an integrated catalytic–immunomodulatory nanoplatform that couples mechanically regulated enzymatic catalysis with immune activation, enabling synergistic tumor therapy.

**FIGURE 5:**
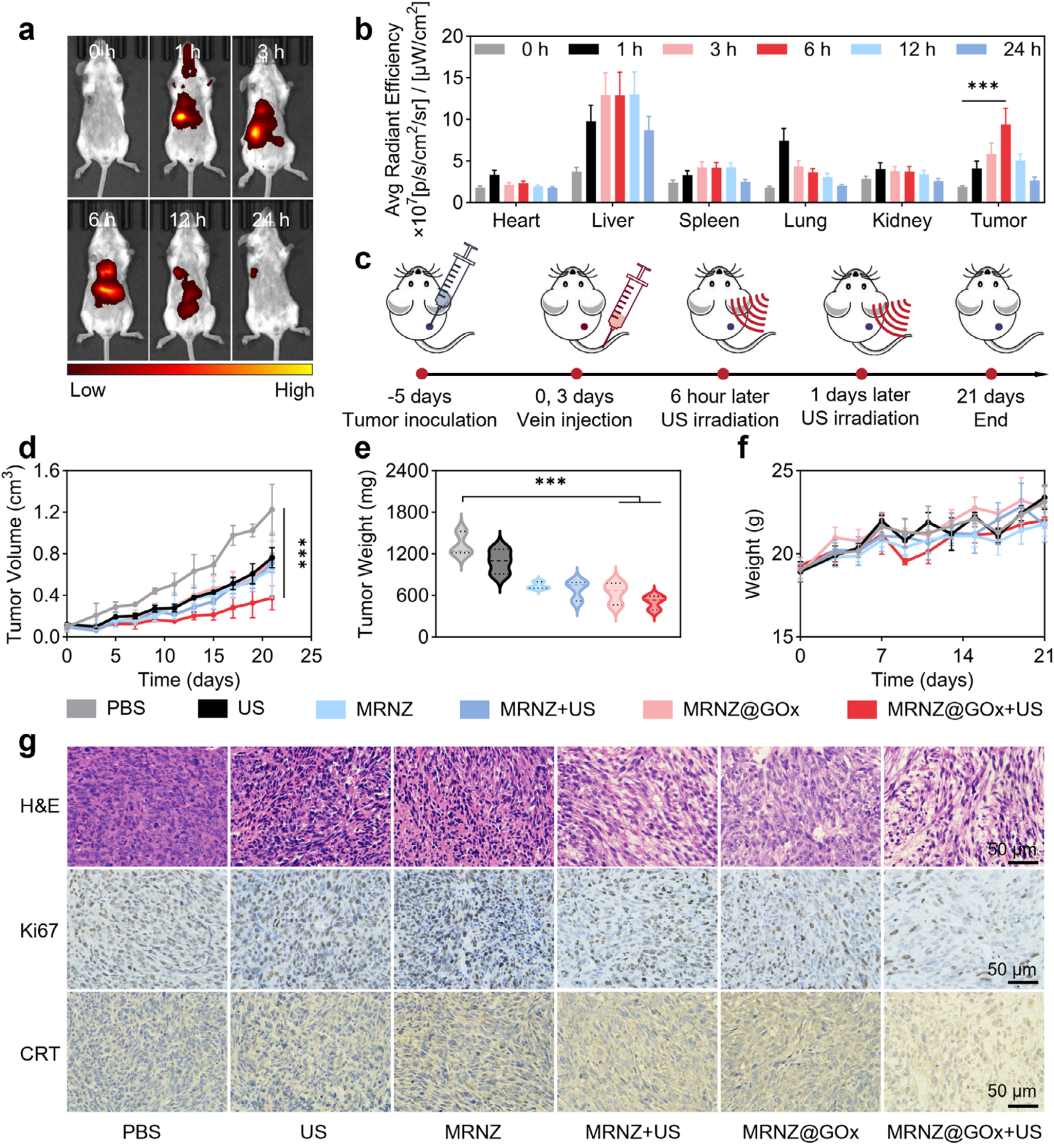
(a) Biodistributions and (b) fluorescence intensity of dissected organs after intravenous administration of Cy5.5‐labeled‐MRNZ@GOx. (c) Illustration of the mechano‐switching triggered cascade catalytic treatment regimen of MRNZ@GOx. (d) Relative tumor volume during different treatment. (e) The average tumor weight after 21 days of treatment. (f) Body weight changes of the mice during different treatments. (g) Representative H&E, Ki67, and CRT staining images of the dissected tumor tissues after 21 days of treatment. Scale bar, 50 µm. Data are mean ± SD (*n* = 5); ***p < 0.001 were assessed via one‐way ANOVA with Tukey's multiple comparison tests.

The biocompatibility and biosafety of MRNZ@GOx were systematically evaluated. Hemolysis assays revealed less than 3.0% hemolysis even at concentrations up to 500 µg/mL (Figure S18), demonstrating excellent blood compatibility. Histological examination of major organs (heart, liver, spleen, lung, and kidney) revealed no detectable pathological abnormalities following treatment (Figure S19). In addition, serum biochemical parameters, including alanine aminotransferase (ALT), aspartate aminotransferase (AST), creatinine (CREA), and blood urea nitrogen (BUN), remained within normal physiological ranges (Figure S20), demonstrating favorable biocompatibility. To assess biodistribution and pharmacokinetics, Cy5.5‐labeled MRNZ@GOx was intravenously injected into tumor‐bearing mice. As shown in Figure 5a, in vivo fluorescence imaging at 6 h revealed substantial retention at the tumor site, while there was expectable accumulation in the liver. A gradual increase in fluorescence intensity was observed in the tumor region, reaching a maximum at approximately 6 h post‐injection (Figures 5b and S21a) We therefore selected it as the optimal time point for the following therapeutic intervention. Specifically, the cascade catalytic activity of MRNZ@GOx could be switched OFF‐ON in a mechanically tunable manner under US irradiation. Such a unique feature might provide a remoted level of spatial and temporal control beyond nanoparticulate accumulation itself, thereby helping to reduce off‐target toxicity.

The in vivo therapy of MRNZ@GOx was evaluated in 4T1 tumor‐bearing mice exposed to US irradiation at 6 h post‐injection (Figure 5c). As shown in Figures 5d‐f and S21b, the MRNZ@GOx + US group exhibited pronounced tumor growth inhibition, achieving a tumor inhibition rate of 69.4%, markedly outperforming all control groups. Importantly, MRNZ@GOx + US treatment caused much more tumor shrinkage than MRNZ + US. This verified the critical contribution of the GOx/POD cascade reaction, which continuously supplies H_2_O_2_ in situ to sustain catalytic activity (Figure 5d). Hematoxylin and eosin (H&E) staining revealed extensive tumor necrosis in the MRNZ@GOx + US group (Figure 5g), while Ki67 immunostaining demonstrated a marked reduction in proliferative activity relative to other groups. Besides, immunohistochemistry revealed much higher CRT exposure on tumor cell membranes in the MRNZ@GOx + US group, matching the activation of ICD (Figure 5g). These results confirmed that US‐triggered cascade catalysis in MRNZ@GOx lead to strong combined tumor inhibition through catalytic therapy and immune system activation.

**FIGURE 6:**
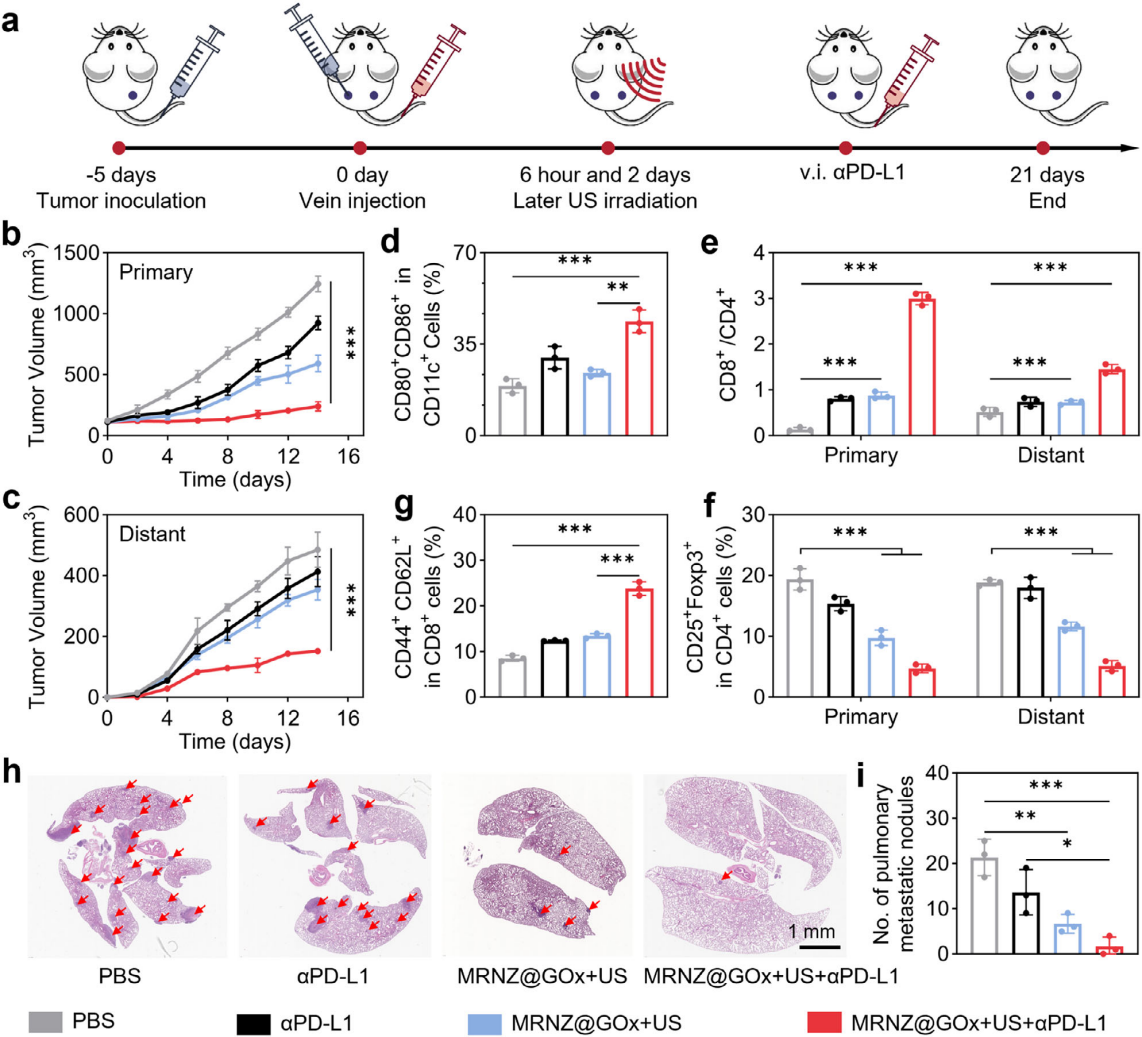
(a) Illustration of the catalytic treatment regimen of MRNZ@GOx combined immune treatment regimen for 4T1 bilateral tumor model. (b) Tumor growth curves of primary tumor. (c) Tumor growth curves of distant tumor. Data are mean ± SD (*n* = 5), ***p < 0.001 were assessed via one‐way ANOVA with Tukey's multiple comparison tests. (d) Expression of CD86^+^ and CD80^+^ in mature DC cells (CD11c^+^MHCII^+^) at lymph. (e) Ratio of CD8^+^/CD4^+^ in bilateral tumor. (f) Percentage of Treg cells (CD25^+^Foxp3^+^) in bilateral tumor. (g) Percentage of TCM cells in spleen. (h) H&E staining images of lung with metastasis nodules (red‐marked). (i) Number of pulmonary metastasis nodules. Data are mean ± SD (*n* = 3); *p < 0.05,**p < 0.01, ***p < 0.001 were assessed via one‐way ANOVA and two‐way ANOVA with Tukey's multiple comparison tests.

To assess the immune‐amplifying potential of MRNZ@GOx, combination therapy with αPD‐L1 immune checkpoint blockade was assessed using a 4T1 bilateral tumor model (Figure 6a). In this model, the primary tumor on the left flank received US irradiation, while the distant tumor on the right flank remained untreated. Notably, mice treated with MRNZ@GOx + US + αPD‐L1 exhibited strong suppression of both primary and distant tumors, much outperforming either monotherapy (MRNZ@GOx + US or αPD‐L1 alone) (Figure 6b,c). To analyze the immune mechanisms, DC maturation and T cell activation were analyzed. The combination treatment induced the highest proportion of mature DCs (CD11c^+^CD80^+^CD86^+^), reaching 43.1% compared with only 19.4% in the PBS control group (Figures 6d and S22). Tumor tissues from the combination group displayed markedly higher number of cytotoxic T lymphocytes (CD8^+^/CD4^+^) (Figures 6e, S23, and S24), showing strong adaptive immune activation. Regulatory T cells (Tregs, CD25^+^Foxp3^+^), which suppress antitumor immunity, were also assessed. As shown in Figures 6f and S25, the proportion of Tregs in the MRNZ@GOx + US + αPD‐L1 group decreased dramatically to 4.85%, compared to 19.2% in the PBS group, suggesting effective immune reprogramming within the TME. Proinflammatory cytokines further showed much higher levels of TNF‐α and IL‐6 in tumors after the combination treatment (Figure S26), confirming immune activation. To check the establishment of long‐term immune memory, spleens were harvested on Day 21 post‐treatment for flow cytometric analysis of memory T cell subsets (Figures 6g and S27). Mice receiving MRNZ@GOx + US + αPD‐L1 treatment exhibited a substantial increase in both effector memory T cells (TEM, CD3^+^CD8^+^CD44^+^CD62L^−^) and central memory T cells (TCM, CD3^+^CD8^+^CD44^+^CD62L^+^), indicating the development of durable antitumor immunity. Importantly, this synergistic catalyst–immunotherapy effectively suppressed lung metastasis (Figure 6h,i), showing the systemic and long‐lasting nature of the immune protection. Histopathological analysis revealed no detectable damage in major organs after the synergistic therapy (Figure S28). Collectively, these results demonstrated that US‐activated MRNZ@GOx effectively elicited antitumor immune responses. When combined with αPD‐L1 checkpoint blockade, this synergistic catalyst‐immunotherapy hybrid platform offered a powerful approach for overcoming immune resistance and achieving sustained tumor regression in solid malignancies.

## 3 Conclusion

The catalytic activity of natural enzymes is finely and dynamically mechano‐regulated through conformational rearrangements. Inspired by this principle, we developed an MRNZ by integrating Fc, a well‐established mechanophore, into a polymeric nanocapsule framework. Incorporation of Fc endowed MRNZ with pronounced mechano‐responsiveness while simultaneously providing abundant reactive sites for efficient catalysis. Upon US irradiation, acoustic shear forces stretch the Fc units, triggering sub‐nanostructural transformations that reduce electron density and relieve steric constraints around the Fe active centers. This process, analogous to natural enzymatic mechanical regulation, enhances electron transfer and optimizes substrate adsorption and product desorption, thereby enabling reversal. As a result, MRNZ exhibits reversible, on‐demand switching of POD‐like activity with exceptional spatiotemporal precision.

As a proof of concept, our work introduced a new approach for ultrasound‐controlled mechanical regulation. This offered a strategy for the precise regulation of nanozyme activity through controllable sub‐nanostructural reconfiguration. Notably, MRNZ fabrication was straightforward, relying on pristine Fc and organic frameworks that afforded strong biocompatibility, regulatory feasibility, and minimal safety concerns. This mechano‐responsive catalytic platform enabled controlled generation of low‐dose •OH that acted as intracellular second messengers, allowing safe and cost‐effective induction of protective oxidative stress responses. To further extend this concept toward therapeutic applications, hollow MRNZ architectures were used to encapsulate auxiliary enzymes such as GOx, yielding an MRNZ@GOx. This design effectively couples GOx‐mediated glucose oxidation with POD‐like catalysis to achieve in situ H_2_O_2_ generation and amplified ROS generation, enabling synergistic cascade catalytic therapy. Importantly, the resulting nanoreactor retained reversible and mechanically tunable enzymatic regulation under US irradiation, allowing efficient yet controlled metabolic reprogramming for tumor catalytic therapy. Overall, our work introduced a MRNZ platform capable of spatiotemporally precise catalytic control for therapeutic modulation. By integrating mechanical responsiveness with enzyme‐like regulation, this strategy bridged the gap between natural mechanoenzymes and mechanically responsive nanozymes, opening new avenues for intelligent catalytic systems in biomedicine.

## Conflicts of Interest

The authors declare no conflicts of interest.

## Supporting information




**Supporting File**: anie72863‐sup‐0001‐SuppMat.docx.

## Data Availability

The data that support the findings of this study are available from the corresponding author upon reasonable request.

## References

[1] Z. Sun , B. Zhang , H. Tu , C. Pan , Y. Chai , and W. Chen , “Advances in Colorimetric Biosensors of Exosomes: Novel Approaches Based on Natural Enzymes and Nanozymes,” Nanoscale 16 (2024): 1005–1024, 10.1039/D3NR05459D. 38117141

[2] V. Tiwari , “In Vitro Engineering of Novel Bioactivity in the Natural Enzymes,” Frontiers in Chemistry 4 (2016): 39, https://www.frontiersin.org/journals/chemistry/articles/10.3389/fchem.2016.00039/full. 27774447 10.3389/fchem.2016.00039 PMC5054688

[3] S. Zhu , Z. Liu , B. Hu , Y. Feng , and G. Pan , “Nitrite Reductases in Biomedicine: From Natural Enzymes to Artificial Mimics,” Research 8 (2025): 0710, https://spj.science.org/doi/full/10.34133/research.0710. 40438154 10.34133/research.0710 PMC12117333

[4] Y. Cheng , Y. D. Xia , Y. Q. Sun , Y. Wang , and X. B. Yin , “‘Three‐in‐One’ Nanozyme Composite for Augmented Cascade Catalytic Tumor Therapy,” Advanced Materials 36 (2023): 2308033, 10.1002/adma.202308033. 37851918

[5] L. H. Fu , Y. Wan , C. Qi , et al., “Nanocatalytic Theranostics With Glutathione Depletion and Enhanced Reactive Oxygen Species Generation for Efficient Cancer Therapy,” Advanced Materials 33 (2021): 2006892, 10.1002/adma.202006892. 33394515

[6] J. Wu , Q. Liu , D. Jiao , et al., “Tensile Strain‐Mediated Bimetallene Nanozyme for Enhanced Photothermal Tumor Catalytic Therapy,” Angewandte Chemie – International Edition 63 (2024): e03203, 10.1002/anie.202403203. 38590293

[7] X. Ma , B. Ding , Z. Yang , et al., “Sulfur‐Vacancy‐Engineered Two‐Dimensional Cu@SnS_2–x_ Nanosheets Constructed via Heterovalent Substitution for High‐Efficiency Piezocatalytic Tumor Therapy,” Journal of the American Chemical Society 146 (2024): 21496–21508, 10.1021/jacs.4c04385. 39073804

[8] W. D. Gao , Y. Q. Mu , L. Xiao , and Y. Xiao , “Designs, Applications, and Future Perspectives of Nanozymes in Orthopedics and Dentistry,” European Cells and Materials 52 (2025): 80–97, https://www.ecmjournal.org/journal/ECM/path/vol052/10.22203/eCM.v052a06.

[9] D. Shao , F. Zhang , F. Chen , et al., “Biomimetic Diselenide‐Bridged Mesoporous Organosilica Nanoparticles as an X‐Ray‐Responsive Biodegradable Carrier for Chemo‐Immunotherapy,” Advanced Materials 32 (2020): 2004385, 10.1002/adma.202004385. 33164250

[10] Z. Wang , F. Chen , Y. Cao , et al., “An Engineered Nanoplatform With Tropism Toward Irradiated Glioblastoma Augments Its Radioimmunotherapy Efficacy,” Advanced Materials 36 (2024): 2314197, 10.1002/adma.202314197. 38713519

[11] F. Chen , H. Huang , F. Zhang , et al., “Biomimetic Chlorosomes: Oxygen‐Independent Photocatalytic Nanoreactors for Efficient Combination Photoimmunotherapy,” Advanced Materials 37 (2024): 2413385, 10.1002/adma.202413385. 39499050

[12] F. Chen , F. Ruan , X. Xie , et al., “Gold Nanocluster: A Photoelectric Converter for X‐Ray‐Activated Chemotherapy,” Advanced Materials 36 (2024): 2402966, 10.1002/adma.202402966. 39044607

[13] Q. Ning , Y. Zhang , H. Sun , et al., “Light‐Harvesting Pigment‐Binding Protein‐Mimicking Carbon Dots for Photodynamic Therapy,” Chinese Chemical Letters 36 (2025): 111133, 10.1016/j.cclet.2025.111133.

[14] J. Peng , F. Chen , Y. Liu , et al., “A Light‐Driven Dual‐Nanotransformer With Deep Tumor Penetration for Efficient Chemo‐Immunotherapy,” Theranostics 12 (2022): 1756–1768, 10.7150/thno.68756. 35198071 PMC8825592

[15] B. Li , Y. Tan , J. H. Lei , et al., “Alkaline Adjuvant Regulates Proteolytic Activity of Macrophages for Antigen Cross‐Presentation and Potentiates Radioimmunotherapy,” Advanced Materials 37 (2025): 2416690, 10.1002/adma.202416690. 39935046 PMC11938008

[16] J. Su , C. B. Musgrave , Y. Song , et al., “Strain Enhances the Activity of Molecular Electrocatalysts via Carbon Nanotube Supports,” Nature Catalysis 6 (2023): 818–828, 10.1038/s41929-023-01005-3.

[17] S. Huo , P. Zhao , Z. Shi , et al., “Mechanochemical Bond Scission for the Activation of Drugs,” Nature Chemistry 13 (2021): 131–139, 10.1038/s41557-020-00624-8. 33514936

[18] Y. Duan , R. Glazier , A. Bazrafshan , et al., “Mechanically Triggered Hybridization Chain Reaction,” Angewandte Chemie International Edition 133 (2021): 20127–20134, 10.1002/ange.202107660. PMC8390435 34242462

[19] G. Liu , A. J. Shih , H. Deng , et al., “Site‐Specific Reactivity of Stepped Pt Surfaces Driven by Stress Release,” Nature 626 (2024): 1005–1010, 10.1038/s41586-024-07090-z. 38418918

[20] V. T. Turitto and C. L. Hall , “Mechanical Factors Affecting Hemostasis and Thrombosis,” Thrombosis Research 92 (1998): S25–S31, 10.1016/S0049-3848(98)00157-1. 9886907

[21] C. Souilhol , J. Serbanovic‐Canic , M. Fragiadaki , et al., “Endothelial Responses to Shear Stress in Atherosclerosis: A Novel Role for Developmental Genes,” Nature Reviews Cardiology 17 (2019): 52–63, 10.1038/s41569-019-0239-5. 31366922

[22] P. H. Chou , S. T. Wang , M. H. Yen , C. L. Liu , M. C. Chang , and O. K. Lee , “Fluid‐Induced, Shear Stress‐Regulated Extracellular Matrix and Matrix Metalloproteinase Genes Expression on human Annulus Fibrosus Cells,” Stem Cell Research and Therapy 7 (2016): 34, 10.1186/s13287-016-0292-5. 26921206 PMC4769486

[23] S. Z. Shirejini , J. Carberry , K. Alt , S. D. Gregory , and C. E. Hagemeyer , “Shear‐Responsive Drug Delivery Systems in Medical Devices: Focus on Thrombosis and Bleeding,” Advanced Functional Materials 33 (2023): 2303717, 10.1002/adfm.202303717.

[24] Z. Chen , R. W. Herzog , and R. J. Kaufman , “Cellular Stress and Coagulation Factor Production: When More Is Not Necessarily Better,” Journal of Thrombosis and Haemostasis 21 (2023): 3329–3341, 10.1016/j.jtha.2023.10.005. 37839613 PMC10760459

[25] H. Itoh , A. Takahashi , K. Adachi , et al., “Mechanically Driven ATP Synthesis by F1‐ATPase,” Nature 427 (2004): 465–468, 10.1038/nature02212. 14749837

[26] M. A. O'Reilly , “Exploiting the Mechanical Effects of Ultrasound for Noninvasive Therapy,” Science 385 (2024): eadp7206, https://www.science.org/doi/abs/10.1126/science.adp7206. 39265013 10.1126/science.adp7206

[27] S. Hao , H. Sheng , M. Liu , et al., “Torsion Strained Iridium Oxide for Efficient Acidic Water Oxidation in Proton Exchange Membrane Electrolyzers,” Nature Nanotechnology 16 (2021): 1371–1377, 10.1038/s41565-021-00986-1. 34697492

[28] Y. Yan , J. Lin , K. Huang , et al., “Tensile Strain‐Mediated Spinel Ferrites Enable Superior Oxygen Evolution Activity,” Journal of the American Chemical Society 145 (2023): 24218–24229, 10.1021/jacs.3c08598. 37874900

[29] Y. Zhang , Z. Wang , T. B. Kouznetsova , et al., “Distal Conformational Locks on Ferrocene Mechanophores Guide Reaction Pathways for Increased Mechanochemical Reactivity,” Nature Chemistry 13 (2020): 56–62, 10.1038/s41557-020-00600-2. 33349695

[30] Y. Yan , J. Zhang , L. Ren , and C. Tang , “Metal‐Containing and Related Polymers for Biomedical Applications,” Chemical Society Reviews 45 (2016): 5232–5263, 10.1039/C6CS00026F. 26910408 PMC4996776

[31] N. Willis‐Fox , E. Rognin , T. A. Aljohani , and R. Daly , “Polymer Mechanochemistry: Manufacturing Is Now a Force to Be Reckoned With,” Chemistry 4 (2018): 2499–2537, 10.1016/j.chempr.2018.08.001.

[32] Z. Jiang , B. B. A. Abbasi , S. Aloko , F. Mokhtari , and G. M. Spinks , “Ultra‐Soft Organogel Artificial Muscles Exhibiting High Power Density, Large Stroke, Fast Response and Long‐Term Durability in Air,” Advanced Materials 35 (2023): e2210419, 10.1002/adma.202210419. 37094185

[33] H. Wu , X. Wang , G. Wang , et al., “Advancing Scaffold‐Assisted Modality for In Situ Osteochondral Regeneration: A Shift from Biodegradable to Bioadaptable,” Advanced Materials 36 (2024): e2407040, 10.1002/adma.202407040. 39104283

[34] C. F. Guimarães , L. Gasperini , A. P. Marques , and R. L. Reis , “The Stiffness of Living Tissues and Its Implications for Tissue Engineering,” Nature Reviews Materials 5 (2020): 351–370, 10.1038/s41578-019-0169-1.

[35] J. Zhang , P. Li , J. Yue , et al., “Gold‐Modified Nanoporous Silicon for Photoelectrochemical Regulation of Intracellular Condensates,” Nature Nanotechnology 20 (2025): 835–844, 10.1038/s41565-025-01878-4. PMC12238954 40234705

[36] S. Dong , S. Liang , Z. Cheng , et al., “ROS/PI3K/Akt and Wnt/β‐Catenin Signalings Activate HIF‐1α‐Induced Metabolic Reprogramming to Impart 5‐Fluorouracil Resistance in Colorectal Cancer,” Journal of Experimental & Clinical Cancer Research 41 (2022): 15, 10.1186/s13046-021-02229-6. 34998404 PMC8742403

[37] P. Y. Wang and J. Harati , “Leveraging Integrative Technologies to Translate Stem Cell and Cell Reprogramming Potential for Neurodegenerative Diseases,” European Cells and Materials 48 (2024): 151–155, https://storage.forummmpub.com/FMP/1968526772808028160/application/v048a09.pdf.

[38] L. Schoenfeldt , P. T. Paine , S. Pico , et al., “Chemical Reprogramming Ameliorates Cellular Hallmarks of Aging and Extends Lifespan,” EMBO Molecular Medicine 17 (2025): 2071–2094, 10.1038/s44321-025-00265-9. 40588563 PMC12340157

[39] Q. Yu , J. Zhou , J. Song , et al., “A Cascade Nanoreactor of Metal‐Protein‐Polyphenol Capsule for Oxygen‐Mediated Synergistic Tumor Starvation and Chemodynamic Therapy,” Small 19 (2022): 2206592, https://onlinelibrary.wiley.com/doi/abs/10.1002/smll.202206592. 10.1002/smll.202206592 36437115

[40] X. Zheng , J. Ma , J. Li , et al., “ATG8ylation‐Mediated Tonoplast Invagination Mitigates Vacuole Damage,” Nature Communications 16 (2025): 6621, 10.1038/s41467-025-62084-3. PMC12274413 40681515

