# Supplementary material for: Mechanically Regulated Nanozymes for Remote Metabolic Reprogramming and Precise Cancer Therapy
Source: Angew Chem Int Ed Engl. 2026 May 26;65(30):e8358731. doi: 10.1002/anie.8358731 (PMC13383197; doi:10.1002/anie.8358731)
Supplement: Supplementary file 1 — Supporting File: anie72863‐sup‐0001‐SuppMat.docx. [file ANIE-65-e8358731-s001.docx]

**Mechanically Regulated Nanozymes for Remote Metabolic Reprogramming and Precise Cancer Therapy**

*Fangman Chen, Xiaochun Xie, Hanyao Huang, Ka Hong Wong,* *Jiying Liu, Hui Fang, Shaowen Wang, Jianfang Cao, Yu Tao, Mingqiang Li, Chao Yang*, Wen Sun*, Dan Shao*, and Yunlu Dai**

F. Chen, Prof. Y. Dai

Cancer Centre and Institute of Translational Medicine, Faculty of Health Sciences, University of Macau, Macau SAR, 999078 China

F. Chen, Prof. Y. Dai

MoE Frontiers Science Center for Precision Oncology, University of Macau, Macau SAR 999078, China

K. H. Wong

State Key Laboratory of Quality Research in Chinese Medicine, Institute of Chinese Medical Sciences, University of Macau, Macau SAR, 999078 China

J. Liu

Department of Chemistry, University of Michigan, Ann Arbor, Michigan, 48109, USA

X. Xie, H. Huang, Prof. D. Shao

State Key Laboratory of Oral Diseases and National Clinical Research Center for Oral Diseases and Department of Oral and Maxillofacial Surgery, West China Hospital of Stomatology, Sichuan University, Chengdu, Sichuan, 610041, China

X. Xie, H. Fang, S. Wang, Prof. D. Shao

School of Medicine, South China University of Technology, Guangzhou, Guangdong 510006, China

Prof. C. Yang

Department of Orthopedics, Center for Orthopedic Surgery, The Third Affiliated Hospital of Southern Medical University, Guangzhou, 510630, China

J. Cao, Prof. W. Sun

State Key Laboratory of Fine Chemicals, School of Chemical Engineering, Dalian University of Technology, Dalian, Liaoning, 116024, China

Prof. Y. Tao, Prof. M. Li

Laboratory of Biomaterials and Translational Medicine, Department of Ultrasound, Center for Nanomedicine, The Third Affiliated Hospital, Sun Yat-sen University, Guangzhou 510630, China

* Corresponding author

E-mail addresses: charisyang@outlook.com (Chao Yang); sunwen@dlut.edu.cn (Wen Sun); stanauagate@outlook.com, shaodan@scu.edu.cn (Dan Shao); yldai@um.edu.mo (Yunlu Dai)

**Experimental**

**1. Materials**

1,1'-Ferrocenedicarboxylic Acid, 1'-ferrocene monocarboxylic acid, ethylenediamine, 2,4,6-trihydroxy-1,3,5-benzenetricarbaldehyde were purchased from Adamas. N-Hydroxysuccinimide and 1-ethyl-3(3-dimethylpropylamine) carbodiimide were purchased from Thermo Scientific. 2',7'-Dichlorodihydrofluorescein diacetate (H2DCFH-DA), Glucose Oxidase (GOx) and Cy5.5 labeled Glucose Oxidase (Cy5.5-GOx) were purchased from Xi’an Qiyue Biology. Tetramethylbenzidine (TMB) was purchased from Macklin. All of reagents were analytical grade. Fetal bovine serum (FBS) and dulbecco's modified Eagle's medium (DMEM) were purchased from Gibco Co., Ltd. 0.25% trypsin-EDTA Antibiotic/antimycotic solution was purchased form Tansoole. Lysotracker Green, Annexin V-FITC/PI Apoptosis Detection Kit, H_2_O_2_ Assay Kit, DNA Damage Assay Kit by γ-H2AX Immunofluorescence and pH probe (BCECF AM) were purchased from Beyotime. Dinitrosalicylic acid (DNS) reagent was purchased from Solarbio. Alexa Fluor 647 Anti-Calreticulin antibody (ab196159) were purchased from Abcam. LPS, GM-CSF and IL-4 were purchased from PeproTech. Anti-CD11c-PE, anti-CD86-APC, anti-CD80-PE-Dazzel594 and anti-MHCII-Alexa Fluro700 were purchased from BD (Shanghai, China). Anti-CD45.2-APC/Cy7, anti-CD3-BV421, anti-CD8a-BV786, anti-CD4-Alexa Fluro700, anti-CD44- PerCP/Cy5.5, anti-CD62L-APC/Cy7, Anti-CD45-BV510, anti-CD8a-BV650, Mouse TNF-α ELISA MAX™ Deluxe Set, INF-γ ELISA MAX™ Deluxe Set and Mouse IL-6 ELISA MAX™ Deluxe Set were purchased from BioLegend, Inc.

**2. Characterization**

Nuclear magnetic resonance (NMR) was carried on NMR spectrometer (Bruker BioSpin AG, AVANCE III HD 400). Fourier Transform Infrared Spectrometer (FTIR) was carried on a Thermo iS50 spectrometer at wavelengths of 400 to 4000 cm^-1^. TEM images were performed using a Talos L120c transmission electron microscope (Thermo Fisher). The surface area and pore size distributions were evaluated and calculated by the Brunauer-Emmett-Teller (BET) and Barrett-Joyner-Halenda (BJH) methods (Micromeritics ASAP 2460). UV-Vis spectra were recorded on an UV 2600 spectrophotometer. The hydrodynamic diameter and zeta potential were characterized with a Mastersizer 2000 (Malvern Instruments Ltd.). Electron paramagnetic resonance (EPR) spectra of the radicals were carried on an EPR spectrometer (Bruker A300) with applying 10 μM 5,5-dimethyl-1-pyrroline-N-oxide (DMPO) as •OH trapper. Flow cytometry analysis of immune cells was recorded using a FACS Celesta flow cytometer (BD Biosciences, USA). Ultrasonic focusing system was purchased from Chongqing Supermicro Medical Equipment Co., Ltd. Fluorescence imaging was pictured on Nikon Ti2-U fluorescence microscope and Leica TCS SP8 confocal laser scanning microscope (CLSM). The mechanical properties were measured by AFM using an atomic force microscope (Bruker Dimension Icon, Bruker,). AFM images were acquired in air medium using the Peak Force Quantitative Nanomechanical (QNM) mode on a Dimension Fast Scan. Cantilevers (RTESPA-150-30 probes) having non-conductive silicon nitride tip with resonant frequency in the range of 2 kHz and nominal force constants of 3.1 N/m were used to image all materials. Young’s modulus of the nanoparticles was calculated using the Sneddon-modified Hertz model. Before every measurement and imaging, the cantilevers were calibrated on a clean sapphire wafer slide to ensure that the deviation of kN was within ± 10%.

**3. Synthesis of mechano‑responsive ferrocene (Fc) monomer (****MFcM)**

MFcM was synthesized according to the previous research.^[1]^ A stirred suspension of ferrocene dicarboxylic acid (719 mg) in freshly distilled and degassed Dichloromethane (CH_2_Cl_2_) (50 mL) under argon. Stoichiometric amounts of O-(Benzotriazol-1-yl)-N,N,N’,N’-tetramethyluronium tetrafluoroborate (TBTU, 5.2 mmol) and Diisopropylethylamine (DIEA, 2.5 mmol, 1.0 mL) were added to the suspension. The resulting slurry was stirred at ambient temperature for 15 minutes (until entire dissolution and then was filtered to remove unreacted materials. The residue was washed with freshly distilled and degassed CH_2_Cl_2_ and the filtrate was cooled to 0 °C under argon. In parallel, charge another flask with 9*H*-Fluoren-9-ylmethyl*N*-(2-aminoethyl) carbamate (EDA) (2.0 equivalents, 5.2 mmol, 1.12 g) and DIEA (2.5 equivalents, 1.0 mL) in CH_2_Cl_2_ (20 mL). After 5 minutes at room temperature, transfer the mixture to the other flask through a stain less steel cannula by using standard Schlenk techniques. The reaction mixture was heated to room temperature and stirred for 4 hours (h) at ambient temperature. The reaction mixture was diluted and washed subsequently with distilled water (50 mL), saturated NaHCO_3_ solution (50 mL), water (50 mL), 0.1M HCl (50 mL) and distilled water (50 mL). The organic layer was dried over MgSO_4_ and the filtered organic layer was dried under reduced pressure. The intermediate products ferrocene-1-EDA-Fmoc-1-EDA-Fmoc (**1**) was obtained. Then, ferrocene-1-EDA-Fmoc-1-EDA-Fmoc (570 mg) was dissolved in CH_2_Cl_2_ (15 mL) under argon. Piperidine (15 mL) was add and stirred at ambient temperature until the reaction reaches completion (approximately 20 minutes). Remove the solvent and triturate the residue with cold methanol. The residue was filtered to remove a white precipitate and reduce the volume of the filtrate. The product was precipitated by the addition of cold CH_2_Cl_2_. the brownish solid was obtained after filtration, which decomposed rapidly in presence of air. The MFcM was purified by chromatography on silica gel (EtOAc/n-hexane 3/1).

**4. Synthesis of mechanically regulated nanozyme (MRNZ)**

MRNZ was prepared by a templating strategy using zeolitic imidazolate framework-8 (ZIF-8) as a sacrificial template. Briefly, ZIF-8 was formed by adding 2-methylimidazole to an aqueous solution of Zn(NO_3_)_2_·6H_2_O at a molar ratio of 48.71:3.36 under vigorous stirring for 1 h. Subsequently, 20 mg of the as-prepared ZIF-8 was dispersed in 18 mL of distilled water, followed by the addition of 0.3 mol of MFcM and 0.2 mol of 2,4,6-trihydroxy-1,3,5-benzenetricarbaldehyde, together with 2 mL of acetic acid. The mixture was stirred for 12 h. The solid product was collected by centrifugation, the supernatant discarded, and the pellet redispersed in 30 mL of ethylenediaminetetraacetic acid (EDTA, 0.5 mg/mL) to remove the ZIF-8 template. The resulting suspension was purified by dialysis against pure water, refreshing the water three times, to afford hollow MRNZ.

A nanocapsule system with the same ferrocene loading, in which ferrocene was covalently linked through only one cyclopentadienyl ligand, was denoted as NP/Fc. The corresponding precursor monomer and nanocapsules were synthesized using the same procedure as described above, except that 1,1'-Ferrocenedicarboxylic acid was replaced with 1'-ferrocene monocarboxylic acid. Nanocapsules without ferrocene were prepared following an analogous procedure, with ferrocene dicarboxylic acid replaced by ethylenediamine.

**5. Synthesis of cascade multiple enzyme regulated nanoreactor (MRNZ@GOx)**

MRNZ@GOx was synthesized following the same protocol as above, except that 10 mg of glucose oxidase (GOx) was included during ZIF-8 formation. The dialysate was collected, and the GOx content was quantified using a Bradford protein assay kit.

**6. Study of •OH Generation and the Relative Enzymatic Activity**

For extracellular •OH generation, 5 µL hydrogen peroxide (30%) and 5 µL TMB 10 mg/mL were dropped into centrifuge tubes, in which 100 µL of 100 μg/mL Fc and MRNZ, respectively. The centrifuge tubes were irradiated ultrasonic (US) with different power for 5 min, the generation of hydroxyl radicals (•OH) was determined by the increased absorbance of ox-TMB at 650 nm.

1 mL of 3.2 mM TMB was applied to monitor the chromogenic reaction (λ = 650 nm) of 100 μg/mL MRNZ upon addition of a series H_2_O_2_. NaAc buffer solution (20 mM, pH = 5.2) was used to fill and fix the final volume to 3 mL. The Michaelis-Menten kinetic curve of MRNZ could be acquired by plotting the respective initial velocities against hydrogen peroxide (H_2_O_2_). The Michaelis-Menten constant (K_m_) and maximal velocity Vmax were calculated via the Lineweaver-Burk plotting.

**7. Measurement of oxygen consumption rates (OCR) and extracellular acidification rates (ECAR)**

1 × 10^5^ MSCs were seeded into XFe24 cell culture plates in complete StemPr^TM^ MSC SFM and incubated at 37 °C overnight in 5% CO_2_. To equilibrate temperature and pH of the detection system, cells were washed with assay StemPr^TM^ MSC SFM and incubated at 37 °C for 1 h in a CO_2_-free incubator before assessment. OCR and ECAR were detected with an Agilent Seahors XFe24 extracellular flux analyzer (Agilent Technologies, USA). To examine mitochondrial respiratory activity, cells were treated with oligomycin (1.5 μM), FCCP (1 μM) and rotenone/antimycin A (0.5 μM) using a Seahorse XF Cell Mito Stress Test Kit. For assessment of glycolytic activity, cells were treated with glucose (100 mM), oligomycin (10 μM) and 2-deoxy-D-glucose (2-DG, 500 mM) using a Seahorse XF Glycolytic Rate Assay Kit in sequence. All experiments were done in seven replicas each time and data expressed as means with standard errors of the mean (SEM).

**8. Glucose uptake**

To quantify glucose uptake, the accumulation of 2-DG was measured using radioactive 2-deoxy-D-[1,2-^3^H]-glucose. Briefly, cells were cultured in 24-well plates and treated with TGF-b1 for 2 h. Then, cells were washed four times with warm PBS at 37 °C and incubated with 1 μCi of 2- deoxy-D-[1,2-^3^H]-glucose in PBS containing 10 μM of 2-DG for 5 min. The reaction was stopped by four washes with cold PBS. Then, 200 μL of 0.4 M NaOH was added to each well to lyze the cells. Cell lysates were transferred into a scintillation vial containing 10 mL of liquid scintillation counter, and the radioactivity was quantified using a scintillation counter. Counts were normalized to protein concentration.

**9. Lactate release assay**

To measure lactate levels secreted into the culture supernatants, 2 × 10^4^ cells per well were seeded into 24-well plates. The medium was refreshed with 1 mL complete StemPr^TM^ MSC SFM medium per well for 24 h after treating with the indicated agents. The next day, culture supernatants were harvested and the lactate concentration was measured by colorimetric assays according to the manufacturer’s protocol of the Lactate Assay Kit. Additionally, protein quantitation was measured by the BCA method and lactate release counts were normalized to total protein concentrations.

**10. RT-qPCR**

RT-qPCR analysis of MSCs gene expression after preconditioned with mechano-enhanced catalysis of MRNZ, RNAs were extracted and purified from different groups of cells using RNeasy Mini Kit (Qiagen, 74104) with the manufactured provided protocol. Quantitative real-time PCR tests were carried out. Primer sequences are listed in Table S1. The reaction mixes were added to the MicroAmp^TM^ EnduraPlate^TM^ Optical 96-Well Clear Reaction Plates with Barcode (Applied Biosystems, 4483354) and then analyze by LightCycler® 96 Real-Time PCR System (Roche, Switzerland).

**Table S1. Primers set for qRT-PCR**

| Gene | Sequence (5’ → 3’) |  |
| --- | --- | --- |
| *Foxo3* | Forward  Reverse | TTGGTGGATCATCAACCCCG  CACTTGGAGAGCTGGGAAGG |
| *Hspa4* | Forward  Reverse | GATTCCATGGGCGTGCATTC  ATGCCCGTTAATCCAGTGGG |
| *p21* | Forward  Reverse | GAGCACAGCTTCTITGCAGCT  CTCAGGTAGACCTTGGGCAG |
| *p53* | Forward  Reverse | CCATGGCCCCTGTCATCTTT  TGAGGGGAGGAGAGTACGTG |
| *IL6* | Forward  Reverse | GCCTTCTTGGGACTGATGCT  AGCCTCCGACTTGTGAAGTG |
| *Gadd45b* | Forward  Reverse | TGAGGGGAGGAGAGTACGTG  GACAGTTCGTGACCAGGAGG |
| *Btg2* | Forward  Reverse | AGGTTTTCAGTAGGGCGCTC  CACCTTGCTGATGATGGGGT |

**11. Senescence-associated** **β-galactosidase assay**

Light fixation was performed on cells plated on glass coverslips using a solution of 3% paraformaldehyde and 0.2% glutaraldehyde in PBS buffer for 5 min. Fixation solution was then removed, wells were washed several times and stained overnight at 37 °C in a CO_2_-free incubator in a solution of 40 mM citric acid/Na phosphate buffer, 5 mM K_4_[Fe (CN)_6_]3H_2_O, 5 mM K_3_[Fe(CN)_6_], 150 mM sodium chloride, 2 mM magnesium chloride, and 1 mg/mL X-gal with a pH of 5.9-6.0. Finally, images were taken using bright-field microscopy, and the proportion of β-Gal-positive cells was then quantified.

**12. Protein extraction and Western blotting**

To extract total proteins, MSCs were lysed in StemPr^TM^ MSC SFM buffer containing a protease inhibitor and a phosphatase inhibitor. In addition, nuclear, cytosolic, and membrane proteins were extracted using a Nuclear Protein Extraction Kit or a Membrane Protein Extraction Kit according to the manufacturer’s instructions. Protein solutions were boiled in 5 × loading buffer at 98 °C for 10 min and then resolved by SDS-PAGE. All the primary antibodies used in the experiment were: anti-PI3K (1:1000, Affinity), anti-Phospho-PI3K (1:1000, Affinity), anti-AKT (1:500, Abcam), anti-Phospho-AKT (1:1000, Abcam), anti-β-catenin (1:5000, Proteintech).

**13. Assessment of enzyme activity and syner****gistic reactions**

We assessed the catalytic activity of glucose oxidase (GOx) by monitoring glucose consumption and H_2_O_2_ production under a variety of conditions, including ultrasound (US) irradiation (1 W, 1 min per well, 50% duty cycle) and acid modulation. The concentrations of glucose and H_2_O_2_ were measured at time points 0, 10, 20, 40, 60, 90, 120, and 180 minutes in reactions containing MRNZ@GOx (100 µg/mL) and glucose (4 mg/mL). Glucose levels were quantified using the DNS reagent, while H_2_O_2_ concentrations were determined using a commercially available H_2_O_2_ assay kit. Prior to assessing reactive oxygen species (ROS) generation in cells, we investigated the capacity of different conditions to produce hydroxyl radicals (•OH) under US irradiation or acidic environments using a TMB oxidation assay. A 1 mL reaction mixture, consisting of TMB (3.2 mM), glucose (4 mg/mL), and various treatments (GOx, MRNZ, MRNZ@GOx) was added to a tube and the mixture was then measured via UV-Vis spectra to track the generation of •OH. The presence of hydroxyl radicals was confirmed by an increase in absorbance of oxidized TMB.

**14. Assessment of pH fluctuation**

To examine pH variations within a buffered environment, an aqueous solution containing GOx, MRNZ, and MRNZ@GOx was introduced into a phosphate-buffered saline (PBS) solution. The mixture was then subjected to continuous ultrasound (US) irradiation (1 W, 50% duty cycle) for 5 minutes. pH measurements were taken every 5 minutes at room temperature using a pH meter (Thermo, USA) to monitor the changes over the course of the irradiation process.

**15. Cellular uptake**

4T1 cells were cultured at 37°C in a humidified and 5% CO_2_ atmosphere with 90% DMEM, 10% FBS, and 1% penicillin-streptomycin medium. 4T1 cells were seeded in a 24-well plate (2 × 10^4^ cells/well) overnight and incubated with MRNZ labeled Cy5.5 at a final Nanoparticle concentration of IC_25_ for 4 h. DAPI and Lysotracker Green was stained before observation under a fluorescence microscope.

**16. Cellular Viability**

4T1 cells (5 × 10^3^/ per well) were seeded into a 96-well plate. After 24 h of culture, cells were treated with fresh medium containing MRNZ@GOx (0, 0.5, 1, 2, 5, 10, 20, 40, 70 and 100 μg/mL), After 2 h of incubation, cells were either irradiated or were not irradiated with US (1 W, 1 min/per well, duty cycle 50 %) and then cultured for another 22 h. After incubation, cells were treated with 100 μL fresh medium and 10 μL CCK-8 was used to investigate the toxicities of the different formulations at an absorbance wavelength of 450 nm.

**17. Evaluating intracellular acidification of 4T1 cells**

The culture conditions for 4T1 cells were previously established, and the cells were treated with MRNZ@GOx at a concentration of 20 μg/mL for 4 h. Prior to ultrasound (US) irradiation (1 W, 1 min per well, 50% duty cycle), the cells were incubated with the pH-sensitive dye BCECF AM at a concentration of 50 nM for 30 minutes. BCECF AM was excited at 488 nm, and fluorescence emission was captured at 500-545 nm using confocal laser scanning microscopy (CLSM). The fluorescence intensity ratio at 535 nm was quantified using a LAXs analyzer, and the pH values were determined by referencing a previously established calibration curve.

**18. Quantum chemical computational details**

**18.1. Detail of density functional theory (DFT) simulations.**

All calculations in this work were performed using Gaussian 09 program package.^[2]^ Full geometry optimizations were performed to locate all the stationary points, using the PBE0 with the def2svp basis for C and H, and SDD basis for Fe.^[3-5]^ Dispersion corrections were computed with Grimme's D3(BJ) method in optimization.^[6]^ Harmonic vibrational frequency was performed at the same level to guarantee that there is no imaginary frequency in the molecules, i.e. they locate on the minima of potential energy surface. Convergence parameters of the default threshold were retained (maximum force within 4.5 × 10^-4^ Hartrees/Bohr and root mean square (RMS) force within 3.0 × 10^-4^ Hartrees/Radian) to obtain the optimized structure. The optimal structure was identified given that all calculations for structural optimization were successfully converged within the convergence threshold of no imaginary frequency, during the process of vibration analysis.

**18.2. Detail of Simulated Raman spectrum**

All–electron DFT calculations have been carried out by the latest version of ORCA quantum chemistry software (Version 6.1.0) ^[7]^. The B3LYP functional ^[8]^ and def2--SVP basis set ^[9]^ were adopted for all geometry optimization and frequency calculations, and the optimal geometry for each compound was determined. The DFT-D3 dispersion correction with BJ--damping ^[10-11]^ was applied to correct the weak interaction to improve the calculation accuracy. Constant external force was applied on the molecule within the EFEI formalism ^[12]^ by pulling on the two defined atoms.

**19. Intracellular ROS Detection**

ROS sensitive probe, 2’,7’-dichlorodihydrofluorescein diacetate (DCFH-DA) was used to determine generation of ROS, which is easily oxidized by intracellular ROS to a green fluorescent substance 2′,7′-dichlorofluorescein (DCF). After medication for 24 h, DCFH-DA (10 µM) was added to the medium for 20 min. The fluorescence microscopic images were acquired using CLSM.

**20. Apoptosis**

4T1 cells were seeded in a 24-well plate (2 × 10^4^ cells/well) overnight and incubated with MRNZ, MRNZ@GOx and GOx with a final NP concentration of IC_25_. After 1 hour incubation, cells were irradiated with US (1 W, 1 min/per well, duty cycle 50%) and incubated for further 4 h. Cells were detached via 0.25% trypsin without EDTA and signed by Apoptosis Detection Kit for flow cytometry.

**21. Immunogenic cell death and Dendritic Cells (DC) maturation *in vitro***

To examine CRT expression, 4T1 cells were incubated in 24 well plates (5 × 10^4^ cells/well) for 24 h. Cells were treated with MRNZ, MRNZ@GOx and GOx for 1 h and then was irradiated with US (1 W, 1 min/per well, duty cycle 50%). After 3 h incubation, 4T1 cells were harvested and then stained with Alexa Fluor 647 Anti-Calreticulin antibody and analyzed by FACS.

To detect DC maturation *in vitro*, BMDCs were obtained from female BALB/c mice and cultured in RPMI-1640 full medium containing 20 ng/mL recombinant mouse granulocyte-macrophage colony-stimulating factor (GM-CSF) and 10 ng/mL recombinant murine interleukin-4 (IL-4). The BMDCs were co-incubated with treated 4T1 cells for 24 h. Finally, BMDCs were harvested and stained with anti-CD11c-PE, anti-MHCII-Alexa Fluro700, anti-CD86-APC and anti-CD80-PE-Dazzel594, and DC maturation was analyzed by FACS.

**22. Hemolytic test**

MRNZ at varying concentrations were introduced into a 2% red blood cell suspension, ensuring consistent volumes across all samples. The resulting mixtures were incubated at 37°C for 24 h. Following incubation, the mixtures were subjected to low-speed centrifugation (e.g., 2000 rpm for 5 minutes) to pellet the cells, and the supernatants were carefully collected. The absorbance of the supernatant at 540 nm was then quantified using a spectrophotometer. The hemolysis rate was calculated using the following equation:

Hemolysis Rate = [OD_(sample)_-OD_(PBS)_]/[ OD_(water)_-OD_(PBS)_] × 100%

where the OD_(PBS)_ consisted of untreated red blood cells, and the OD_(water)_ represented fully the deionized water-treated red blood cells.

**23. *In vivo* experiments**

Female BALB/c mice (6 weeks old) were obtained from Hunan SJA laboratory animal co., LTD. All protocols for animal studies conformed to the Guide for the Care and Use of Laboratory Animals and the procedures (ACE2023061) were approved by the South China University of Technology Animal Care and Use Committee. Mice were housed in a standard pathogen-free (SPF) -grade pathogen-free facility with a 12 h light/dark cycle (7:00-19:00 light and 19:00-7:00 dark) at 20 ± 3°C and a relative humidity of 40% to 70% for a week prior to experiments.

4T1 breast cancer tumor-bearing mice were established via plant 1 × 10^6^ 4T1 cells/per mice under breast fat pad. 4T1 tumor-bearing mice were randomly divided into different groups (n = 6) when tumor volumes reached 100 mm^3^. Mice were treated with MRNZ, MRNZ@GOx and GOx once every three days. 4T1 tumor-bearing mice were irradiated with US (1 W, 5 min, duty cycle 50 %) after vein injection for 4 h. The αPD-L1 was injected after 24 h. Tumor volume and body weight were evaluated every 2 days. Tumor volume was calculated by the formula, V = 0.5 × L × W × W, where L is the longest dimension and W is the shortest dimension.

To conduct immunological evaluations, BALB/c mice were sacrificed at day 6 post-treatment to collect tumor and serum. The tumor was cut into small pieces and ground in a mouse lymphocyte separation liquid to form a cell suspension, which was then filtered through a 70-μm Falcon cell strainer. 1 mL of DMEM medium was slowly added to 4 mL of the above cell suspension, which was then centrifuged at 800 g for 30 min. Afterward, the intermediate lymphocyte layer was separated, washed, and centrifuged. For analysis of CTLs (CD3^+^CD4^-^CD8^+^) and CD4^+^ T cells (CD3^+^CD4^+^CD8^-^), lymphocytes were stained with anti-CD45.2-APC/Cy7, anti-CD3-BV421, anti-CD4-Alexa Fluro700 and anti-CD8a-BV786 antibodies. Finally, cells were analyzed by FACS. For measurement of pro-inflammatory cytokines, TNF-α, IFN-γ, IL-6 level was detected in serum and tumor samples were made into tissue homogenate was analyzed by ELISA. Lymph nodes were taken to make a single-cell suspension. DC analysis was performed with Anti-CD11c-PE, anti-CD80-D549, anti-MHCII-AF700 and anti-CD86-APC.

To conduct immunological memory, BALB/c mice were sacrificed at day 21 post-treatment to collect spleen. Spleen were taken to make a single-cell suspension and separated Immune memory cell. Immune memory cell was performed with Anti-CD45-BV510, anti-CD3-BV421, anti-CD8a-BV650, anti-CD4-Alexa Fluro700, anti-CD44-PerCP/Cy5.5 and anti-CD62L-APC/Cy7.

**24. *In vivo* evaluation of tumor targeting**

Female BALB/c mice (6 weeks old) were obtained from Hunan SJA laboratory animal co., LTD. 4T1 breast cancer tumor-bearing mice were established via plant 1 × 10^6^ 4T1 cells/per mice under breast fat pad. To observe the biodistribution of different formulations *in vivo*, MRNZ labeled Cy5.5 were intravenously injected into 4T1 tumor-bearing mice. The biodistribution was observed at different time points using the IVIS Lumina imaging system.

**25. Statistical analysis**

All data are shown as means ± standard errors of the mean (SEM). Differences between groups were analyzed by Student’s t-test when comparing only two groups. Differences among more than two groups were analyzed by one-way or two-way analysis of variance (ANOVA), and the Bonferroni post hoc test was used to analyze differences between any two groups. ^*^*P* < 0.05, ^**^*P* < 0.01, ^***^*P* < 0.001 was considered a statistically significant difference.


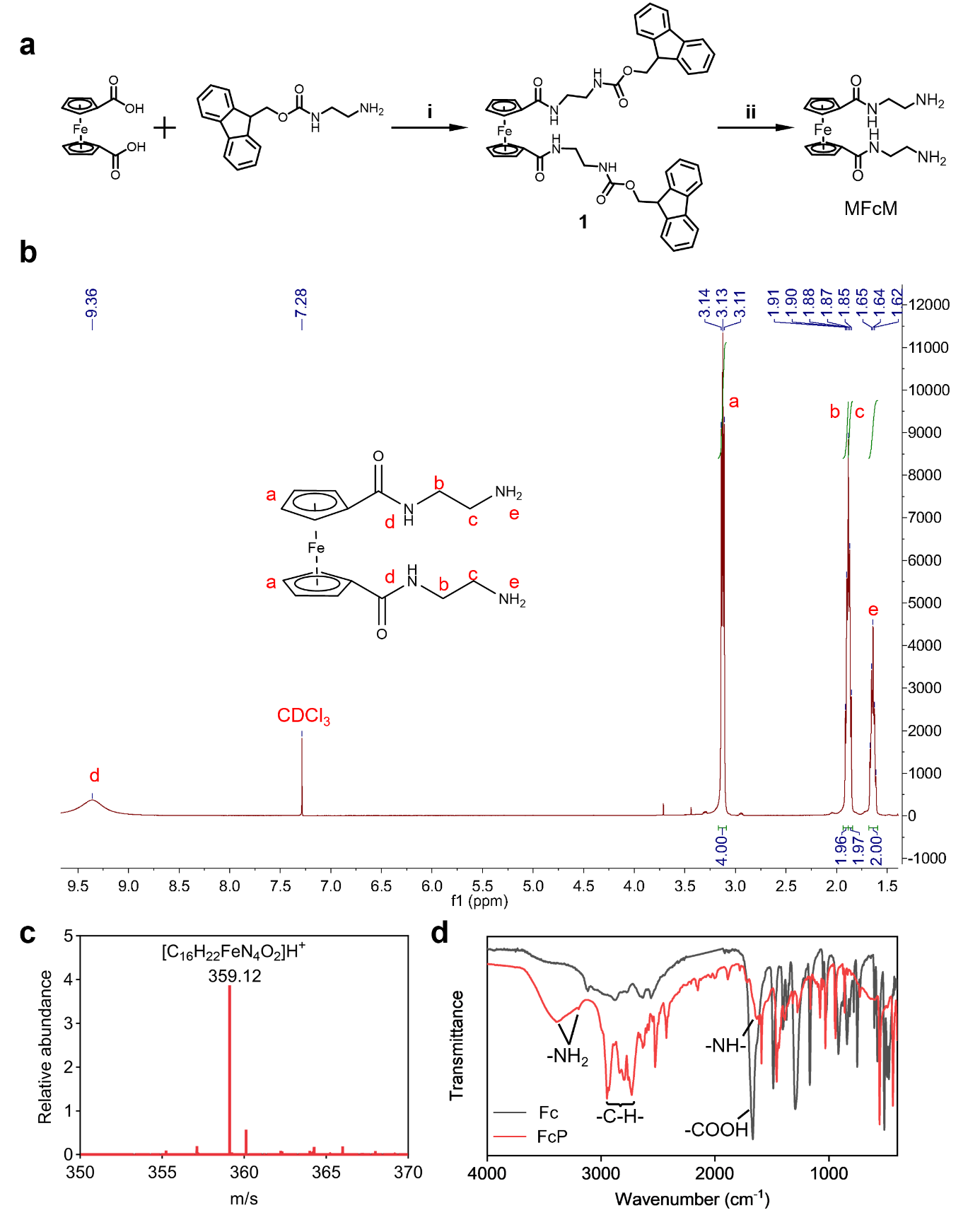


**Figure S1**. Characterization of mechano‑responsive ferrocene (Fc) monomer (MFcM). (a) Synthetic reaction path of MFcM. (b) ^1^H NMR spectra of mechano-responsive ferrocene-based precursor (FcP). ^1^H NMR (400 MHz, CDCl_3_) δ 9.36 (br,1H), 3.13 (t, 4H), 1.90 (m, 2H), 1.87 (m, 2H), 1.64 (m, 2H). (c) Mass spectrometry (MS) of MFcM. (d) Fourier transform infrared spectroscopy of MFcM.


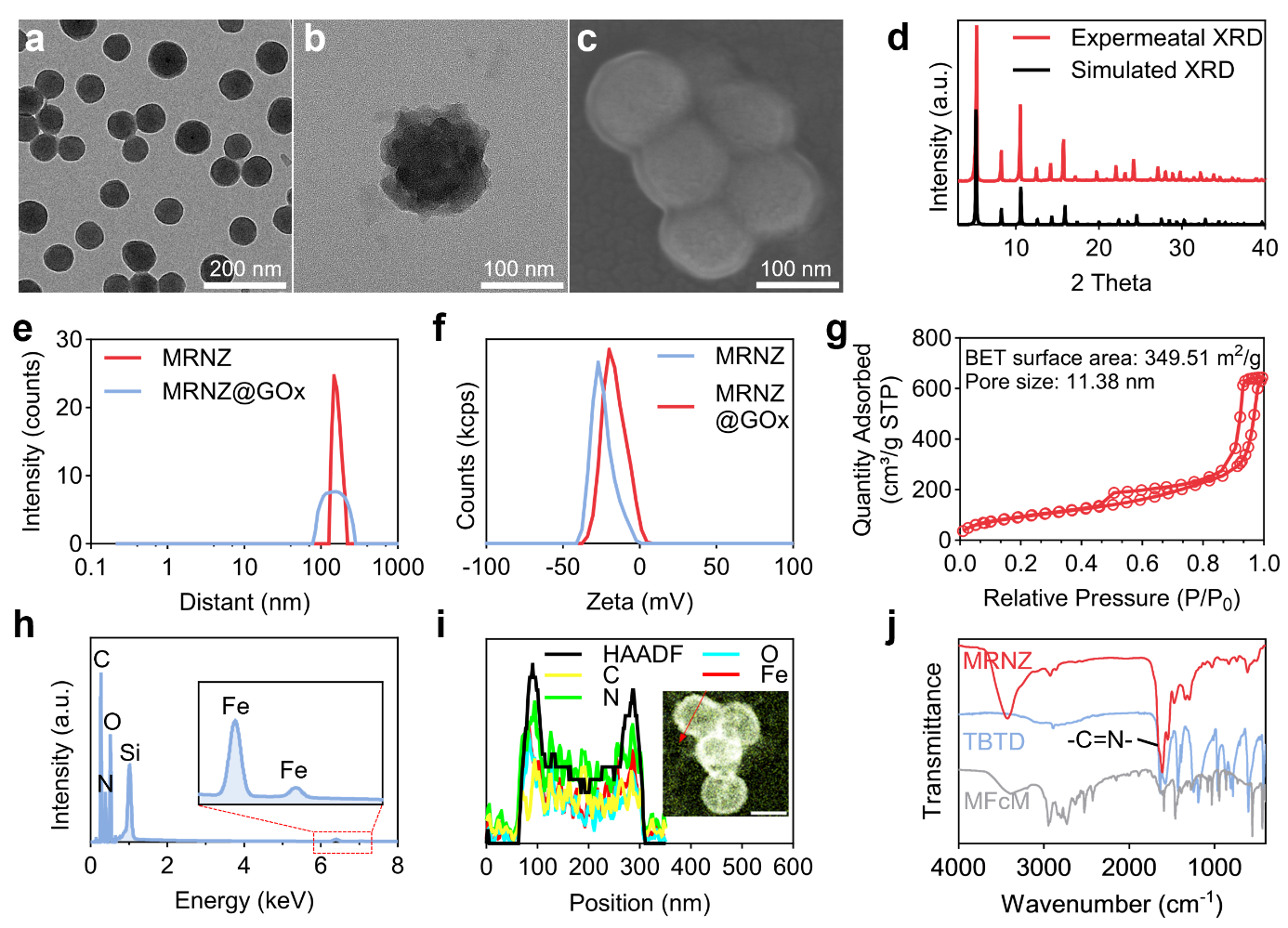


**Figure S2**. Characterization of morphology and components of MRNZ. (a) TEM image of ZIF-8. (b) TEM image of MRNZ with ZIF-8 template. (c) SEM image of MRNZ. (d) XRD pattern of ZIF-8. (e) Hydrodynamic diameter of MRNZ and MRNZ@GOx. (f) Zeta potential of MRNZ and MRNZ@GOx. (g) Nitrogen adsorption-isotherm of MRNZ. (h) SEM-EDS of MRNZ. (i) The HAADF-STEM and EDX-mapping of MRNZ. (j) Fourier transform infrared spectroscopy of MRNZ.


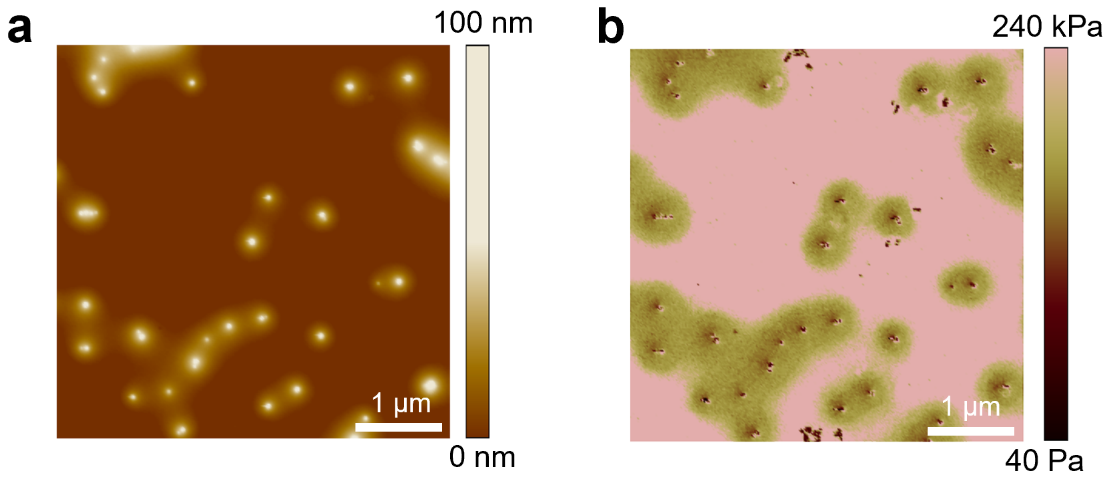


**Figure S3**. (a) AFM height profiles of MRNZ. Scale bars, 1 μm. (b) AFM Young’s modulus profiles of MRNZ. Scale bars, 1 μm.


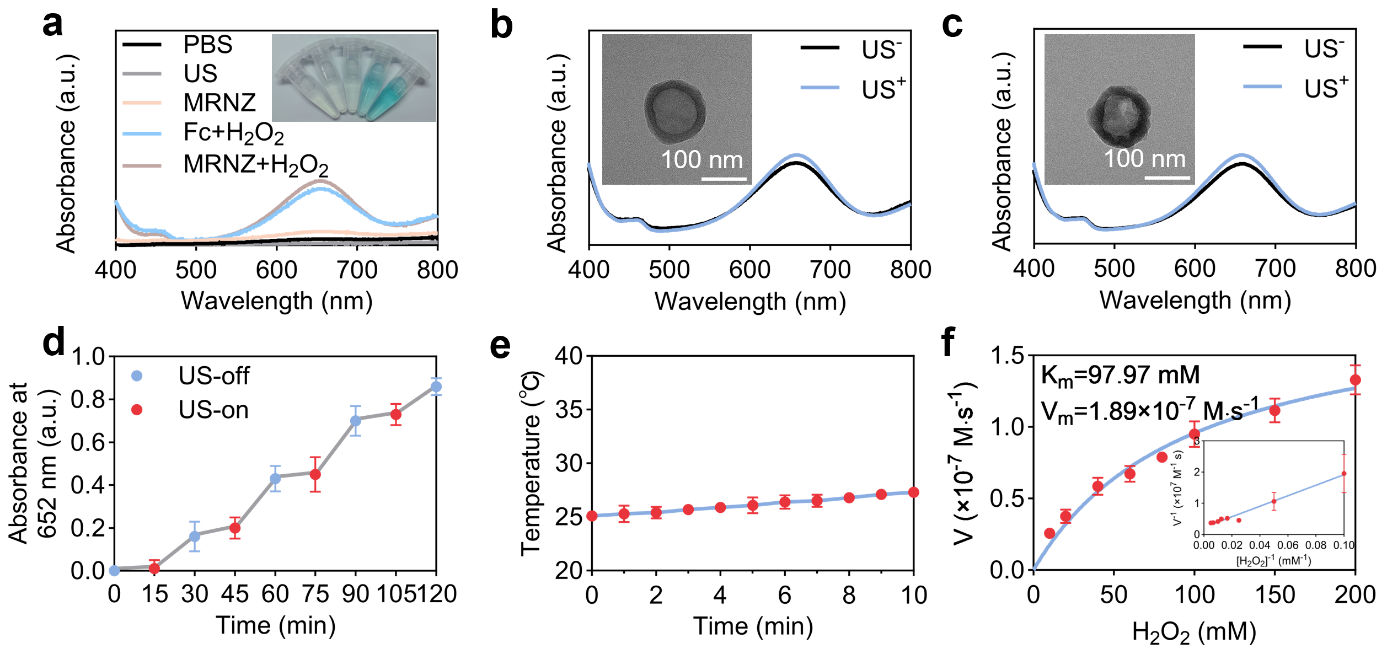


**Figure S4**. (a) UV absorption spectra of TMB with different treatment. (b) UV absorption spectra of TMB with NP + Fc treatment. (c) UV absorption spectra of TMB with NP/Fc treatment. (d) Absorption of TMB oxidation at 652 nm through interval US irradiation. (e) Temperature as a function of US time. (f) The POD-like of MRNZ without US. Typical Michaelis-Menten curves with various concentrations of H_2_O_2_ in NaOAc-HOAc buffer (100 mM, pH 6.5), illustration: the Lineweaver-Burk fitting (double reciprocal) of Michaelis-Menten fitting Curve activity. Data are mean ± SD (n = 3).


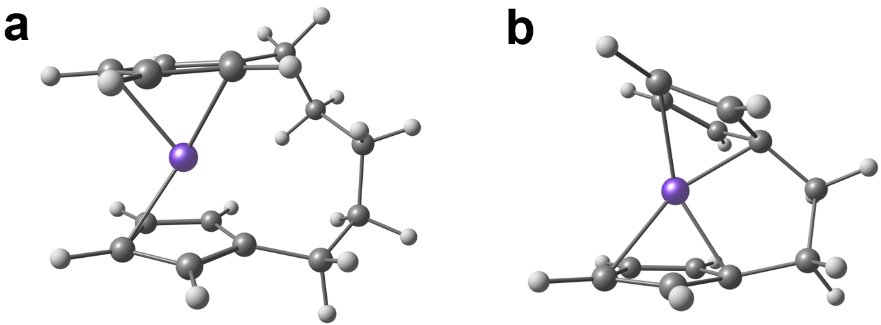


**Figure S5**. The molecular structure of Fc (a) and isomerous Fc (b).


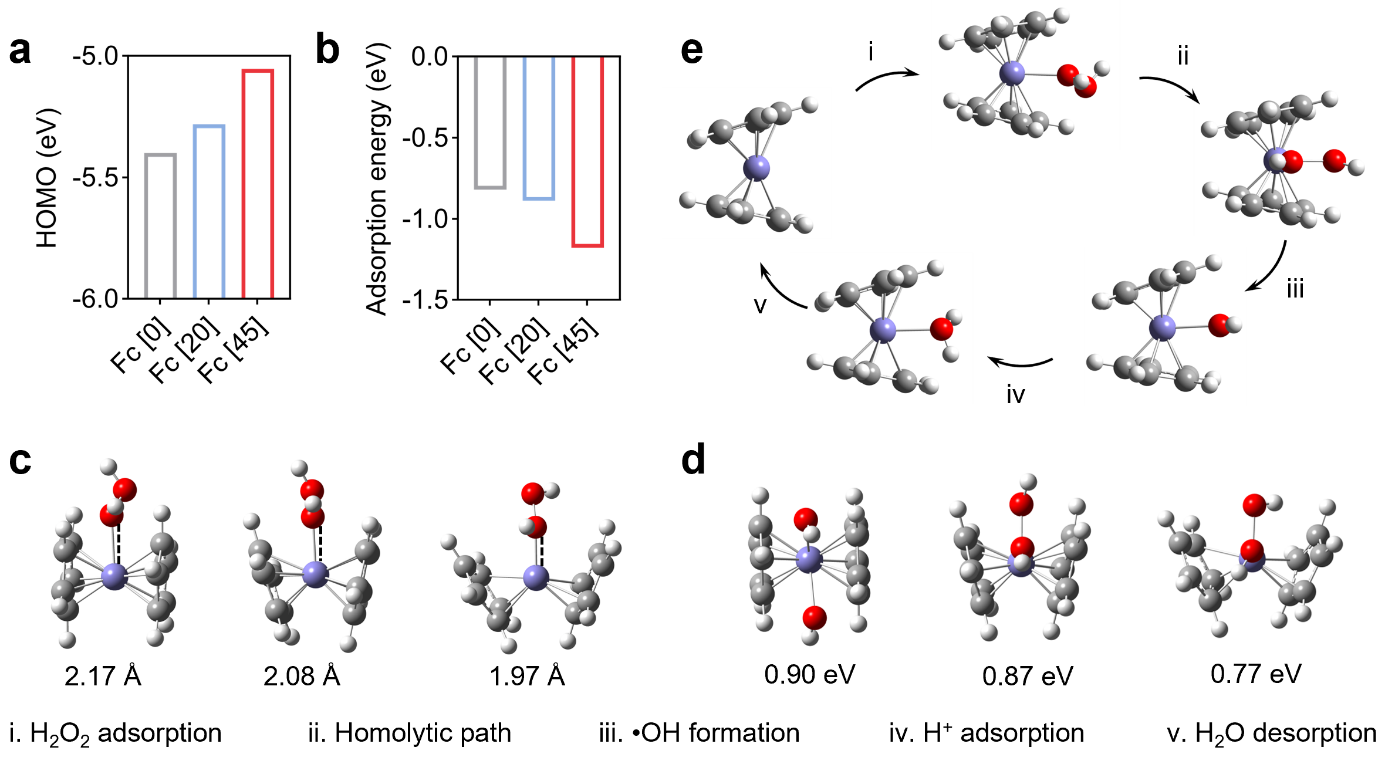


**Figure S6**. (a) HOMO of Fc structure with different angle transition. (b) H_2_O_2_ adsorption energy of Fc structure with different angle transition. (c) The calculated bond length of Fe-O after the H_2_O_2_ adsorption on Fe-Cp site with different angle transition. (d) Dissociation energy of Fc, α = 0° (left), α = 20° (middle), α = 45° (right). (e) Proposed catalytic mechanism for Fenton reaction of Fc (α = 20°).


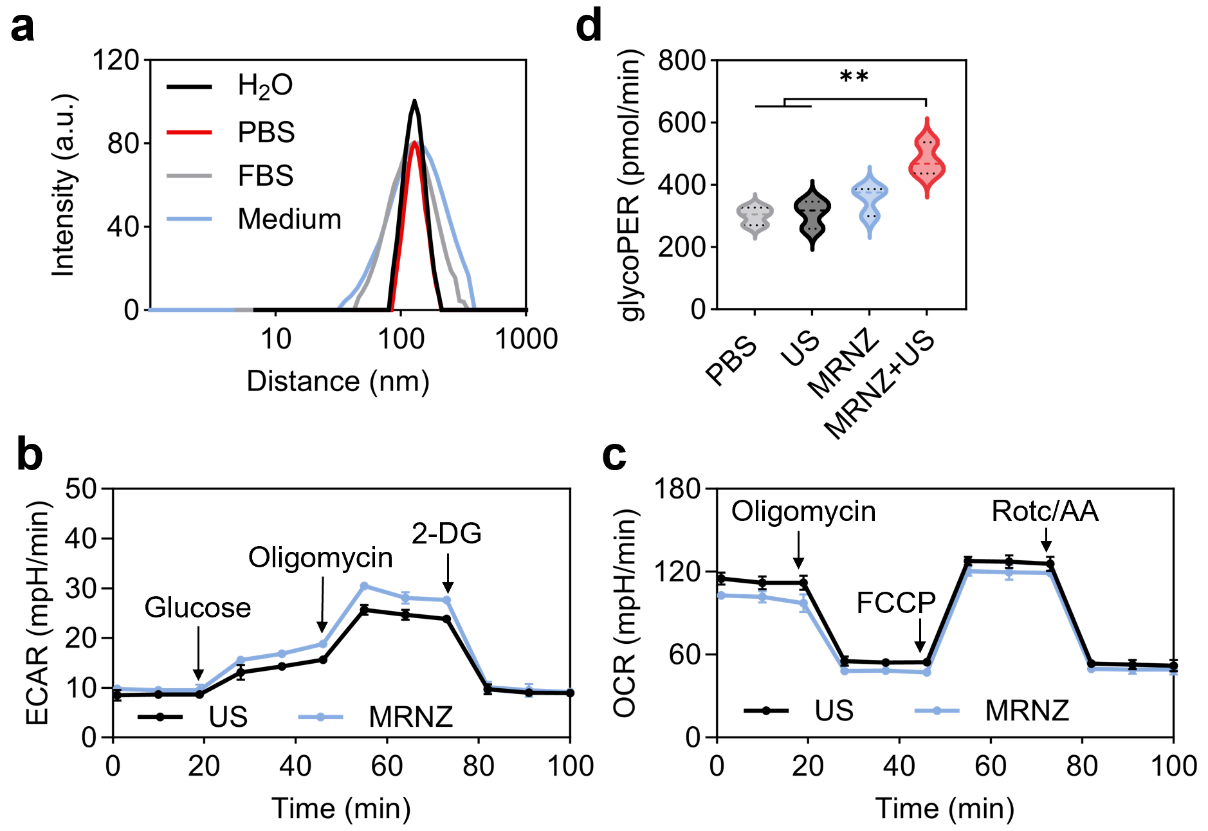


**Figure S7**. (a) Hydrodynamic diameter of MRNZ in different solution. (b) MSCs were treated with different treatment and glucose (100 mM), oligomycin (10 μM) and 2-DG (500 mM) were successively added to measure ECAR. (c) MSCs were treated with different treatment oligomycin (1.5 μM), FCCP (1 μM) and rotenone/antimycin A (0.5 μM) were successively added to measure OCR. (d) Glycolytic capacity of MSCs with different treatment. Data are mean ± SD (n = 3), ***P* < 0.01 were assessed via one-way ANOVA with Tukey’s multiple comparison tests.


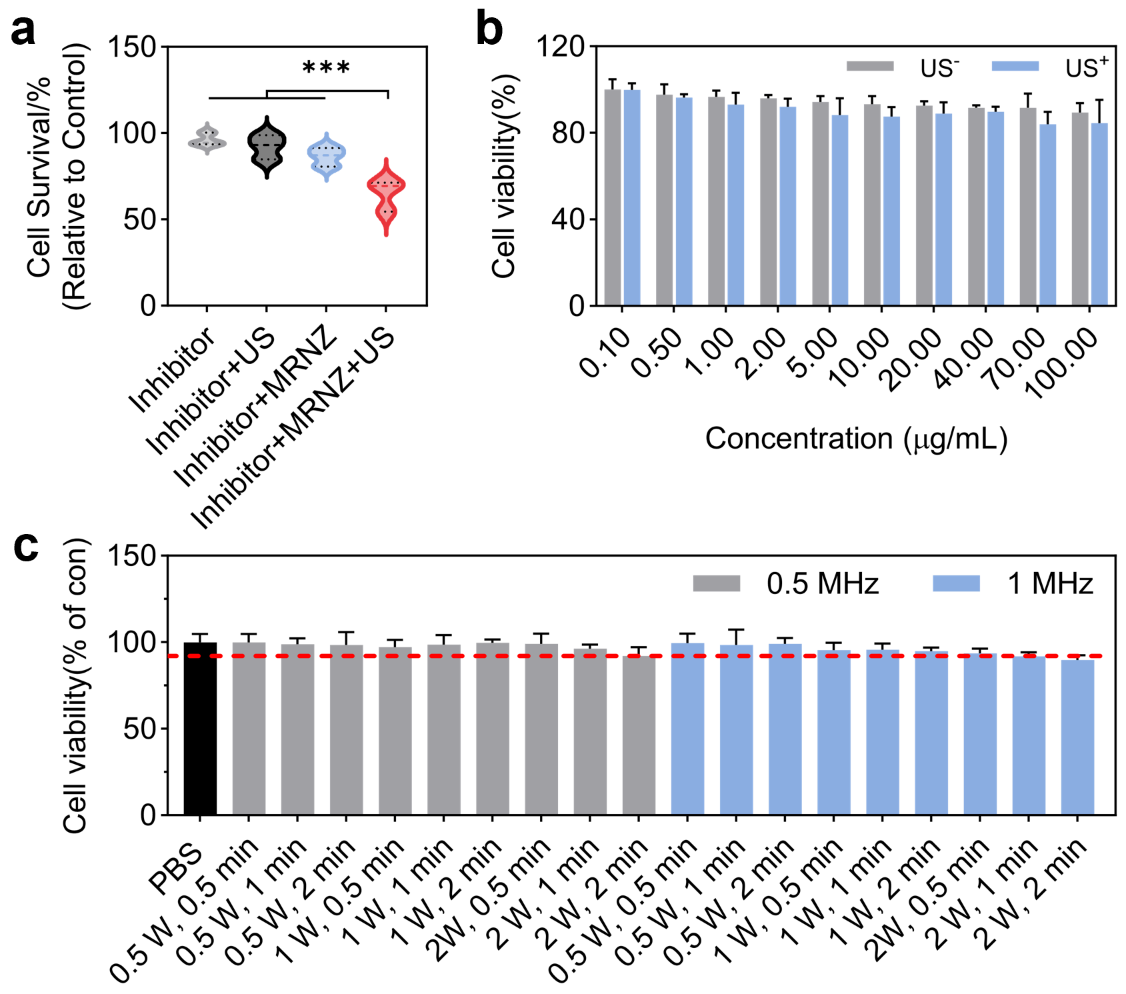


**Figure S8.** (a) MSCs cytotoxicity following treatment and exposure to 50 μM 3-BrPA for 48 h. (b) Cell viability was measured by CCK-8 assays on treatment with different concentration of MRNZ for 48 h. (c) Cell viability in 4T1 cells under different US treatment, red line: Cell viability of 90%. Data are mean ± SD (n = 3), ****P* < 0.001 were assessed via one-way ANOVA with Tukey’s multiple comparison tests.


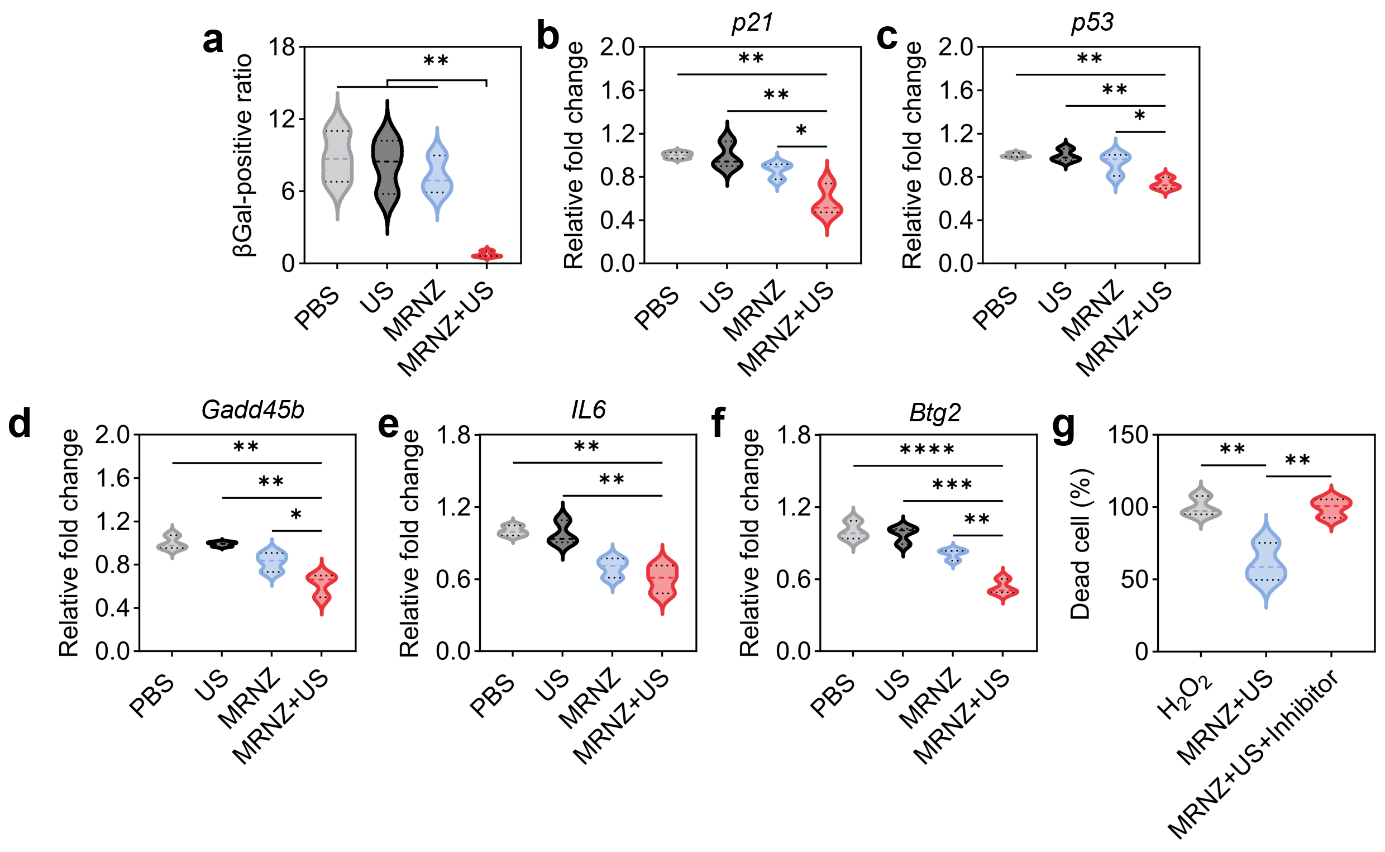


**Figure S9.** (a) Quantification of Senescence-associated beta-galactosidase (SA-beta-gal) staining following different treatment. mRNA levels of senescence-associated and age-related stress response genes (b) *p21*, (c) *p53*, (d) *Gadd45b*, (e) *IL6* and (f) *Btg2* following treatment. (g) MSCs viability following pretreatment with 30 min, 10 μM inhibitor HY-15727 before preconditioning (15 min, MRNZ with US) and 200 μM H_2_O_2_ exposure for 1 h. Data are mean ± SD (n = 3), **P* < 0.05, ***P* < 0.01, ****P* < 0.001, *****P* < 0.0001 were assessed via one-way ANOVA with Tukey’s multiple comparison tests.

**
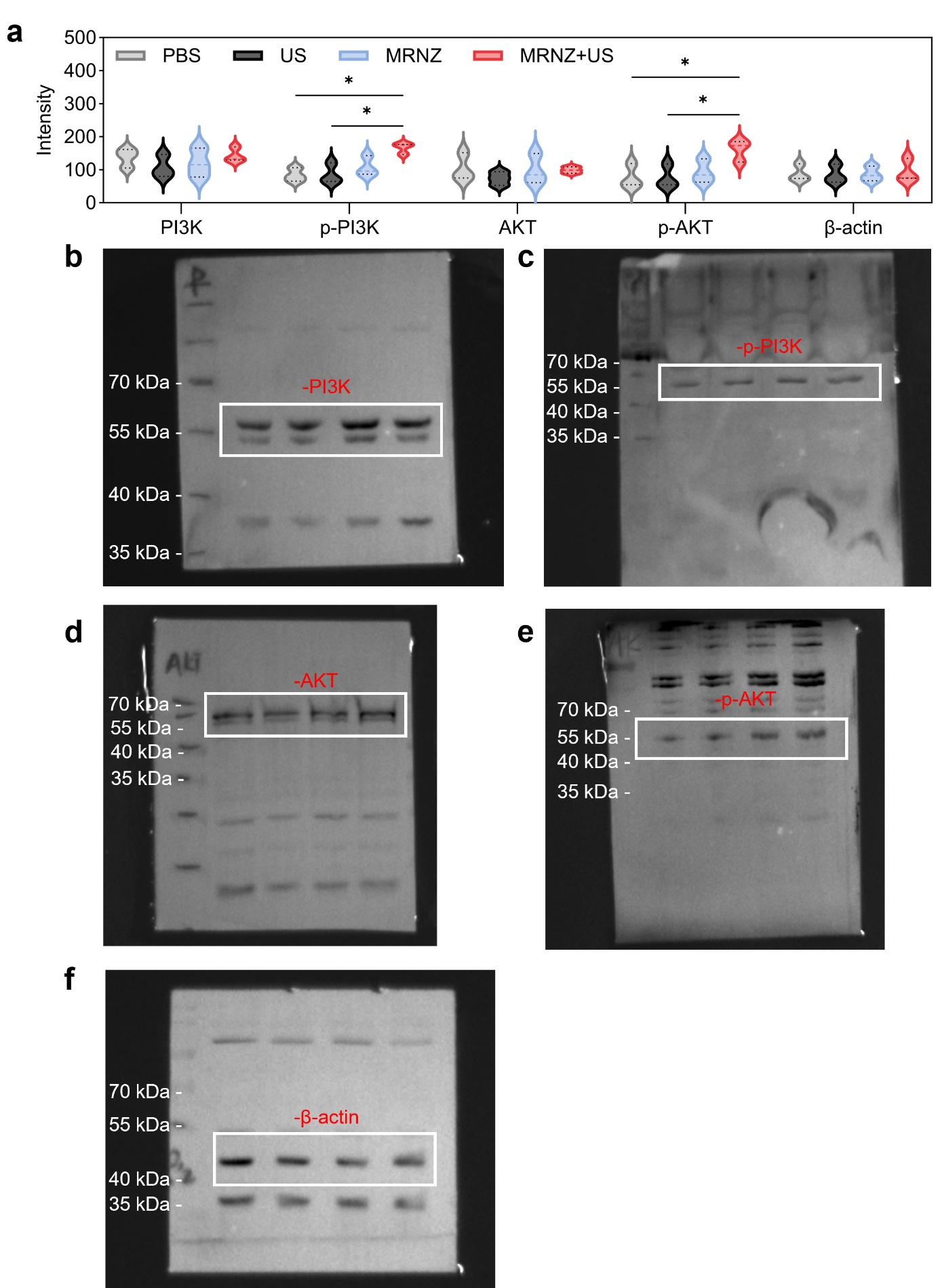
**

**Figure S10**. Quantification of PI3K/AKT pathway members in western blots (a) and uncropped western blot images (b-f). Data are mean ± SD (n = 3), **P* < 0.05 were assessed via two-way ANOVA with Tukey’s multiple comparison tests.


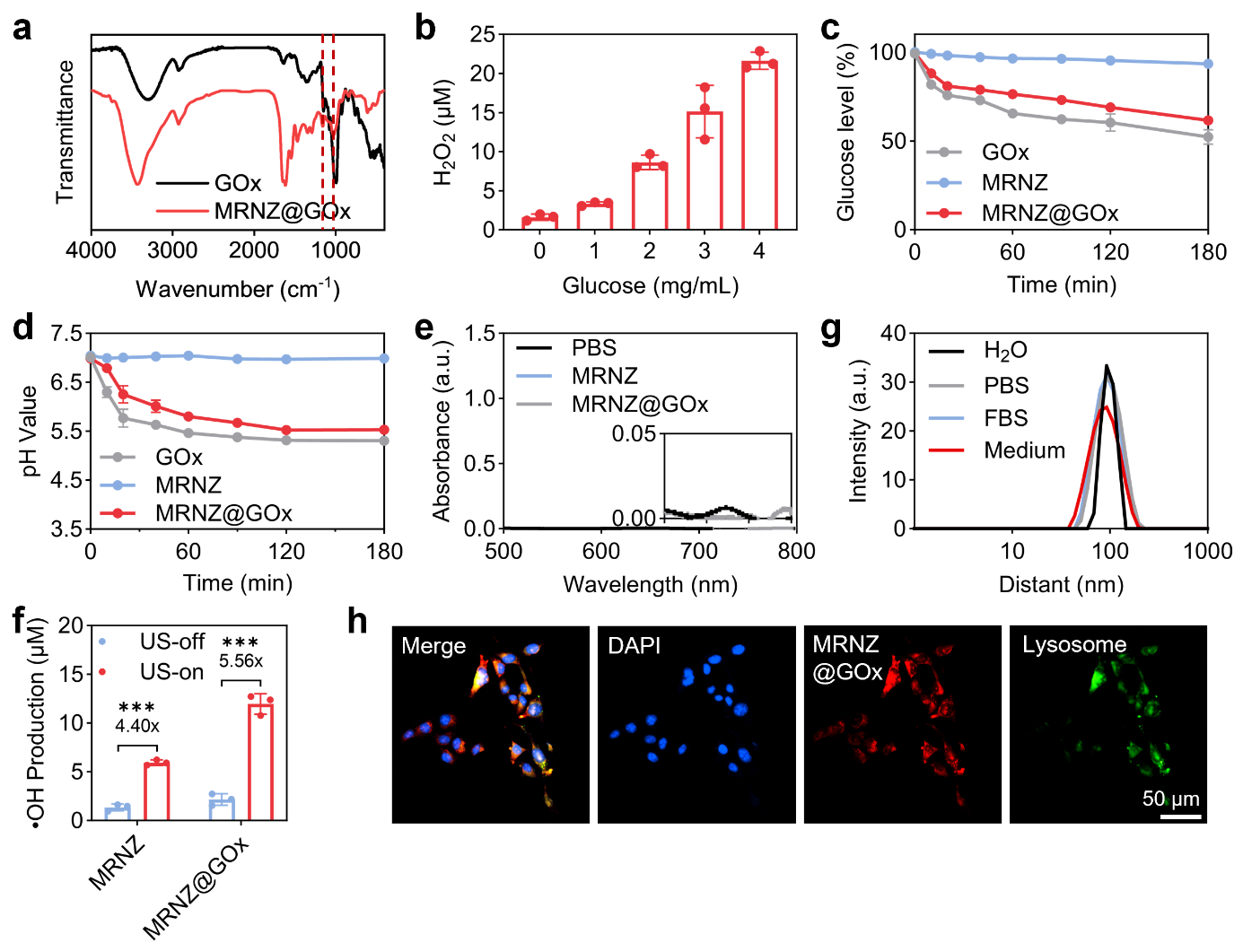


**Figure S11.** (a) Fourier transform infrared spectroscopy of GOx and MRNZ@GOx. (b) Glucose content-dependent H_2_O_2_ generation. (c) Variation in glucose depletion in cell culture media after the addition of GOx, MRNZ and MRNZ@GOx. (d) pH value variation from GOx, MRNZ and MRNZ@GOx. (e) UV absorption spectra of TMB with PBS, MRNZ and MRNZ@GOx treatment with US without glucose. (f) POD-like activity of MRNZ@GOx for •OH production. (g) Hydrodynamic diameter of MRNZ@GOx in different solution. (h) Fluorescent microscopy images of cell take up MRNZ@GOx. Data are mean ± SD (n = 3), ****P* < 0.001 were assessed via one-way ANOVA with Tukey’s multiple comparison tests.


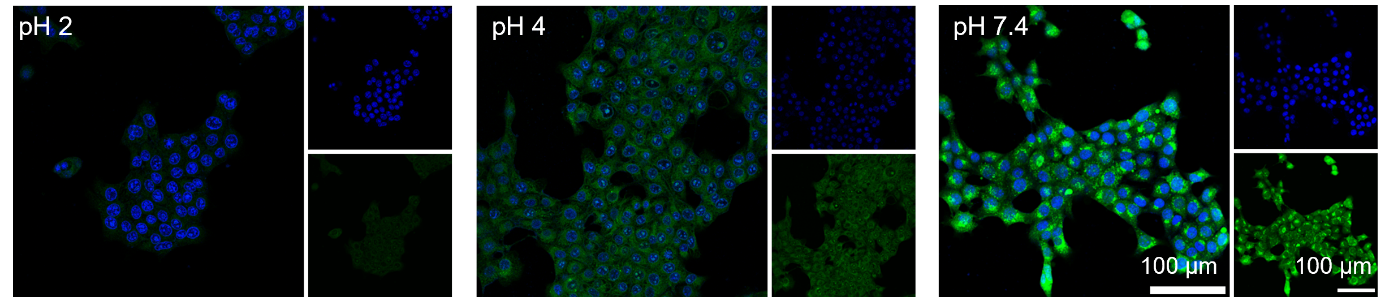


**Figure S12.** Confocal fluorescent microscopy images of pH probe (BCECF AM) in 4T1 incubated in PBS with pH.


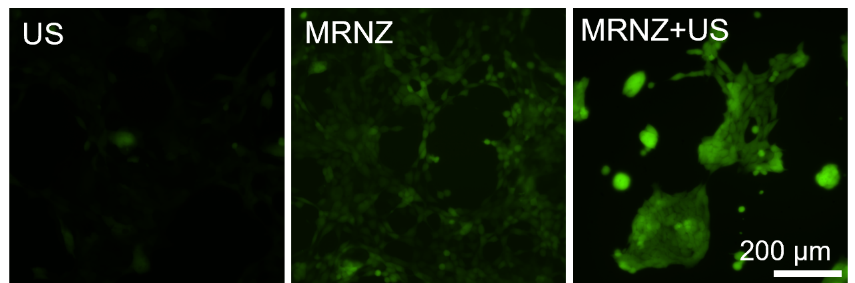


**Figure S13.** Fluorescent microscopy images of H2DCF-DA (green) in treated 4T1 cells.


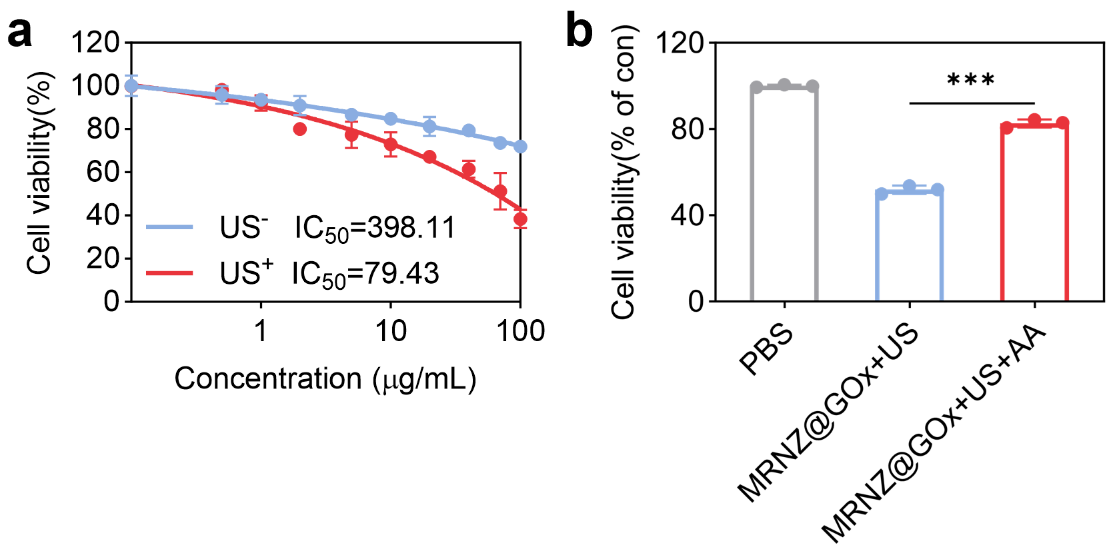


**Figure S14.** (a) Cytotoxicity effects of MRNZ@GOx in 4T1 cells. (b) Cytotoxicity effects of MRNZ@GOx in 4T1 cells with the addition of L-ascorbic acid. Data are mean ± SD (n = 3), ****P* < 0.001 were assessed via one-way ANOVA with Tukey’s multiple comparison tests.


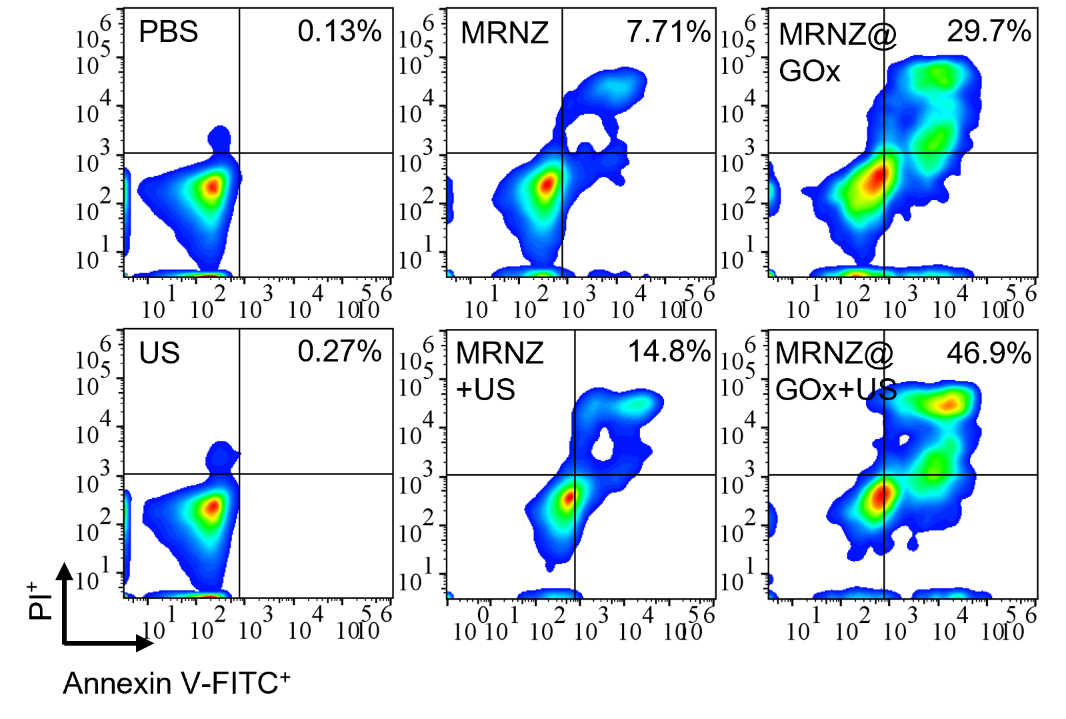


**Figure S15.** Flow cytometric analysis of apoptosis in 4T1 cells with different treatment.


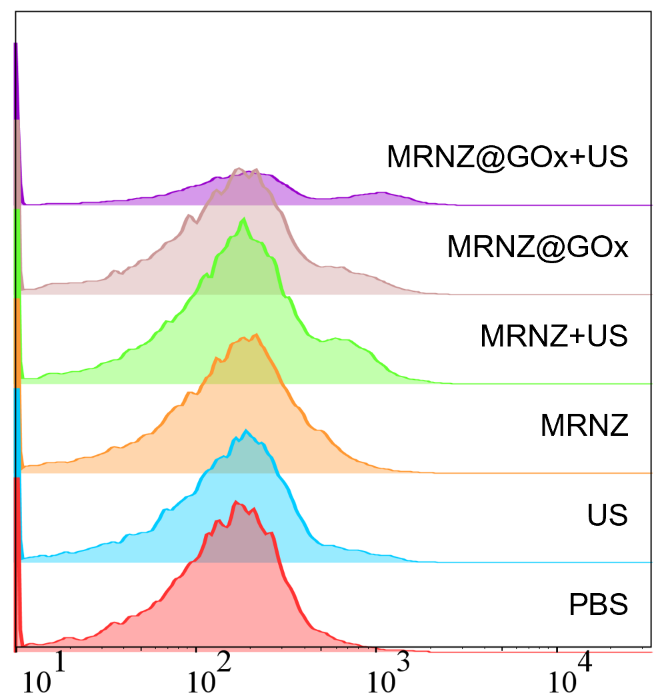


**Figure S16.** Flow cytometric analysis of CRT eversion degrees in 4T1 cells under different treatments.


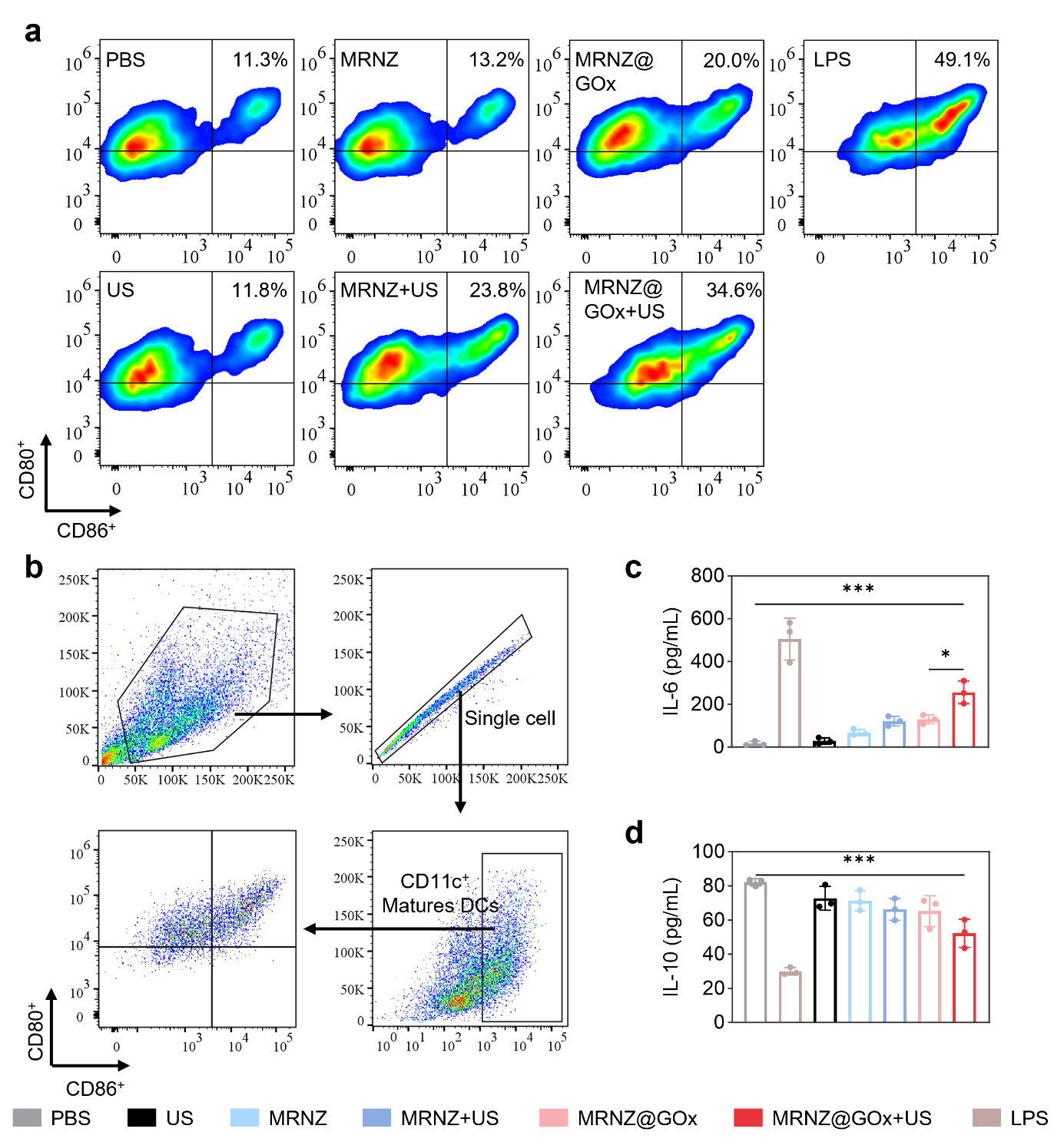


**Figure S17.** (a) Percentage of CD80^+^CD86^+^ expression in mature DCs (CD11c^+^MHCII^+^) after coincubation with treated 4T1 cells by flow. (b) Representative flow cytometry gating strategies for APC cells. (c) IL-6 levels in the supernatant of bone marrow-derived dendritic cells (BMDCs) after coincubation with treated 4T1 cells. (d) IL-10 levels in the supernatant of BMDCs after coincubation with treated 4T1 cells. Data are mean ± SD (n = 3), **P* < 0.05, *** *P* < 0.001 were assessed via one-way ANOVA with Tukey’s multiple comparison tests.


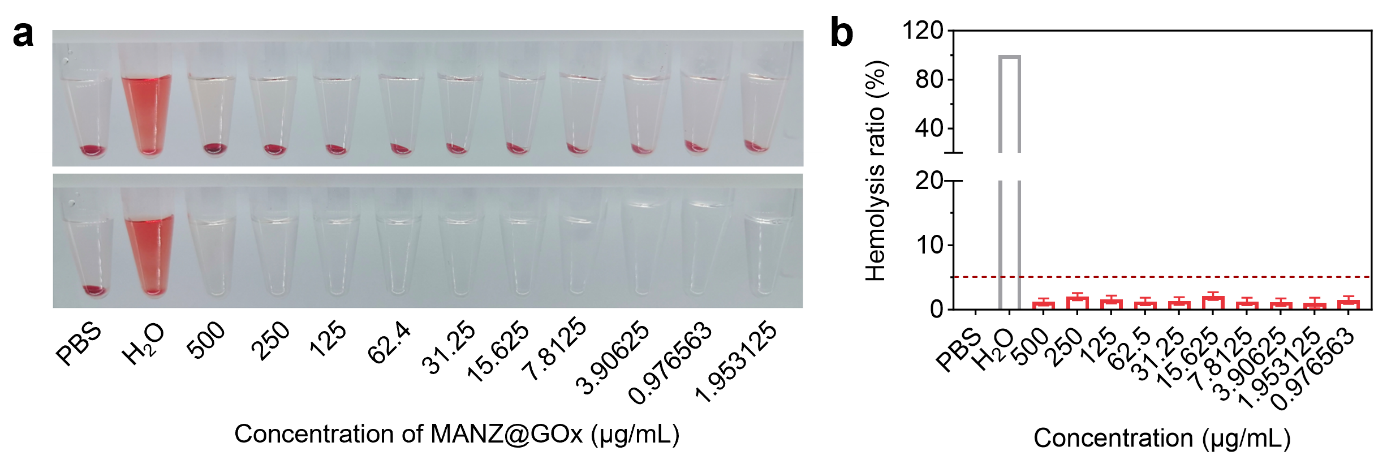


**Figure S18.** Hemolysis tests of MRNZ@GOx on red blood cells. Data are mean ± SD (n = 3), ****P* < 0.001 were assessed via one-way ANOVA with Tukey’s multiple comparison tests.


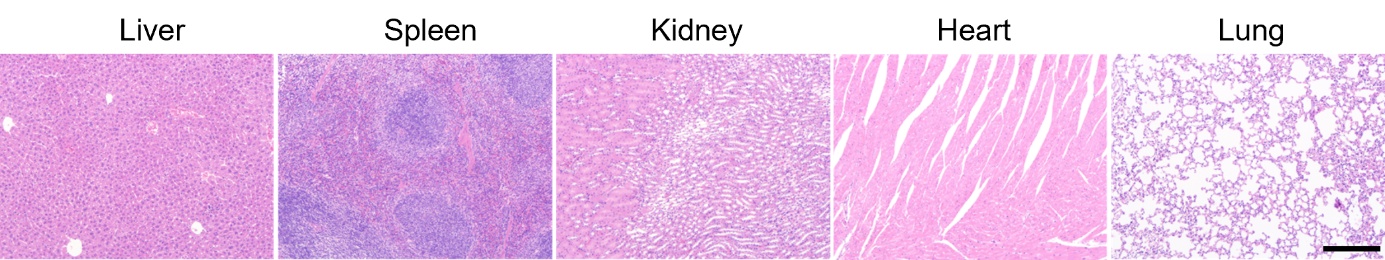


**Figure S19.** H&E staining images of liver, spleen, kidney, heart, and lung of MRNZ@GOx at 14 days. Scale bars, 200 μm.


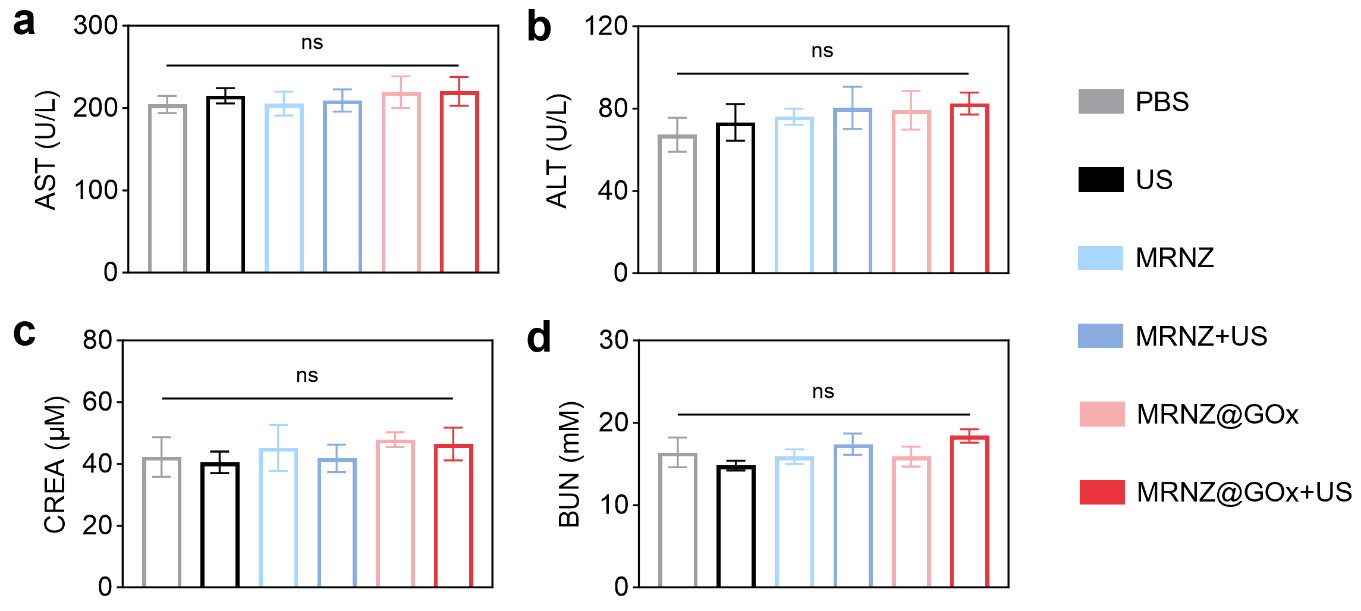


**Figure S20.** Biochemical indicator of blood serum. The levels of AST (a), ALT (b), CREA (c) and BUN (d) from the mice after different treatment. Data are mean ± SD (n = 3).


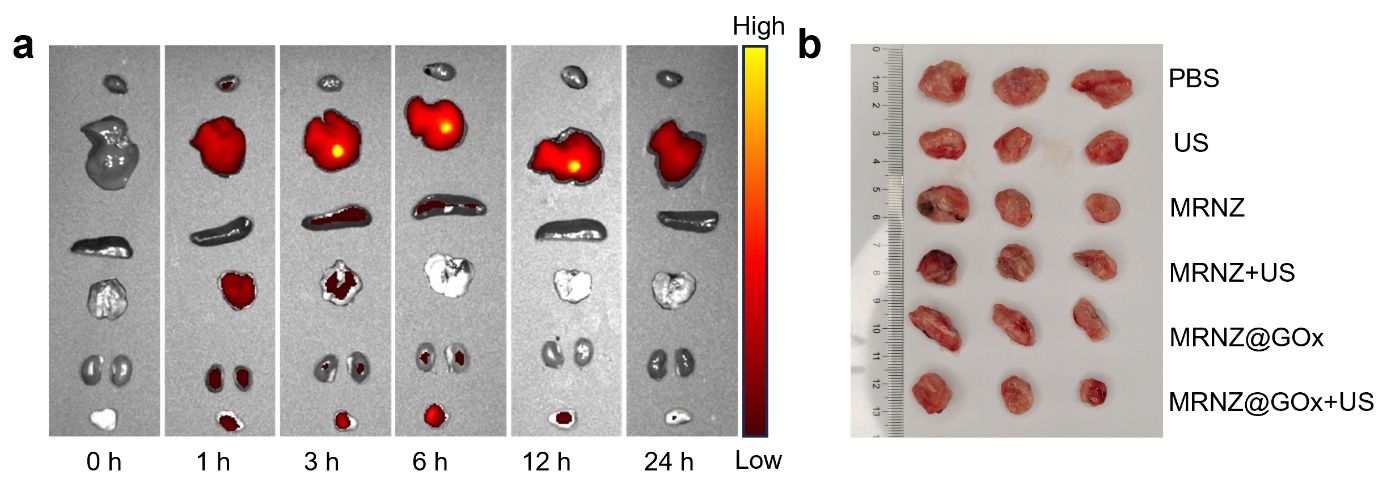


**Figure S21.** (a) Representative fluorescence images of dissected organs and tumors at 0, 1, 3, 6, 12 and 24 h after vein injection of Cy5.5 labeled-MRNZ@GOx. (b) Representative digital photos of excised tumor from the mice after 21 days of treatment.


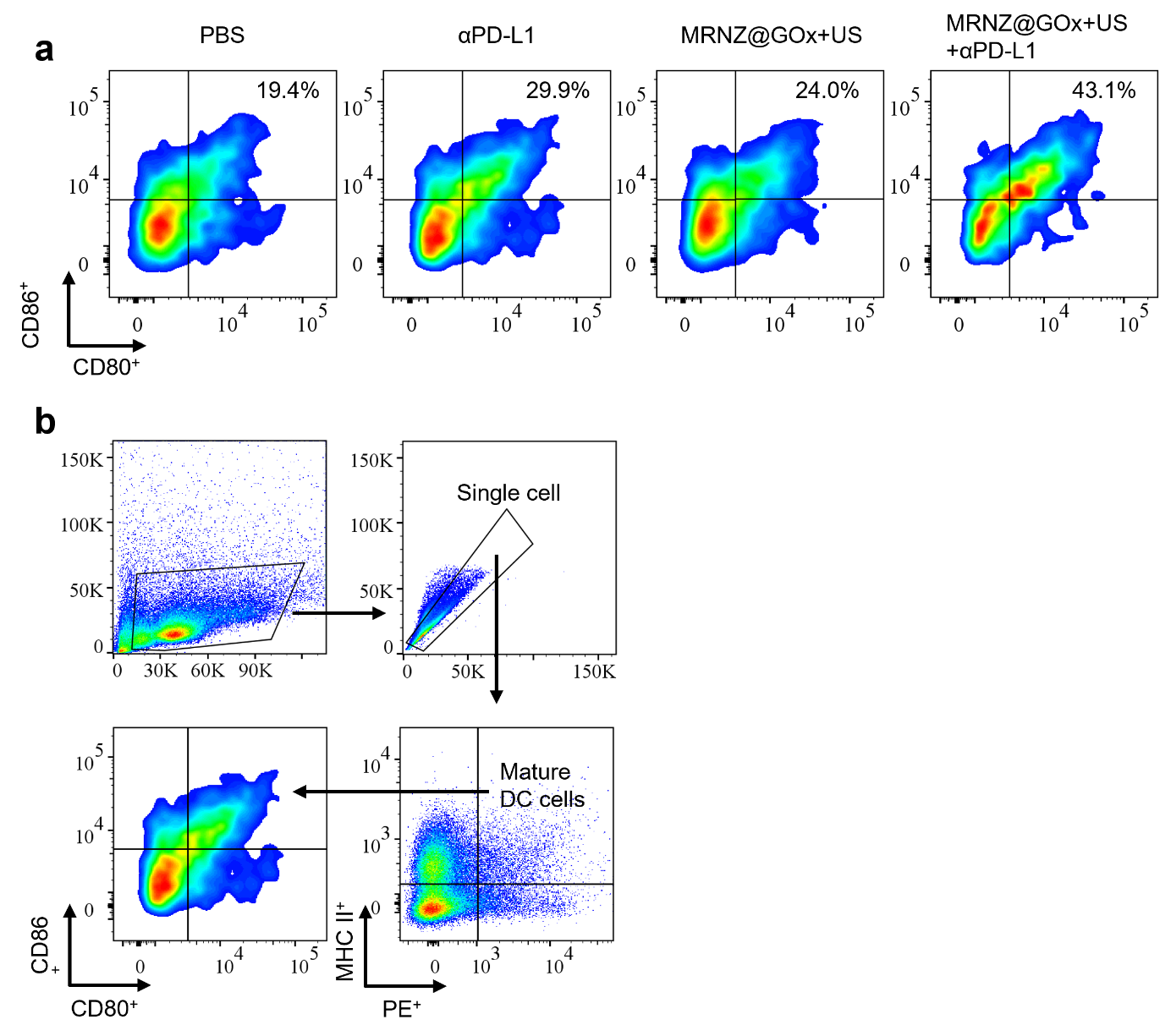


**Figure S22.** (a) Percentage of CD80^+^CD86^+^ expression in mature DCs (CD11c^+^MHCII^+^) at lymph by flow. (b) Representative flow cytometry gating strategies for APC cells.


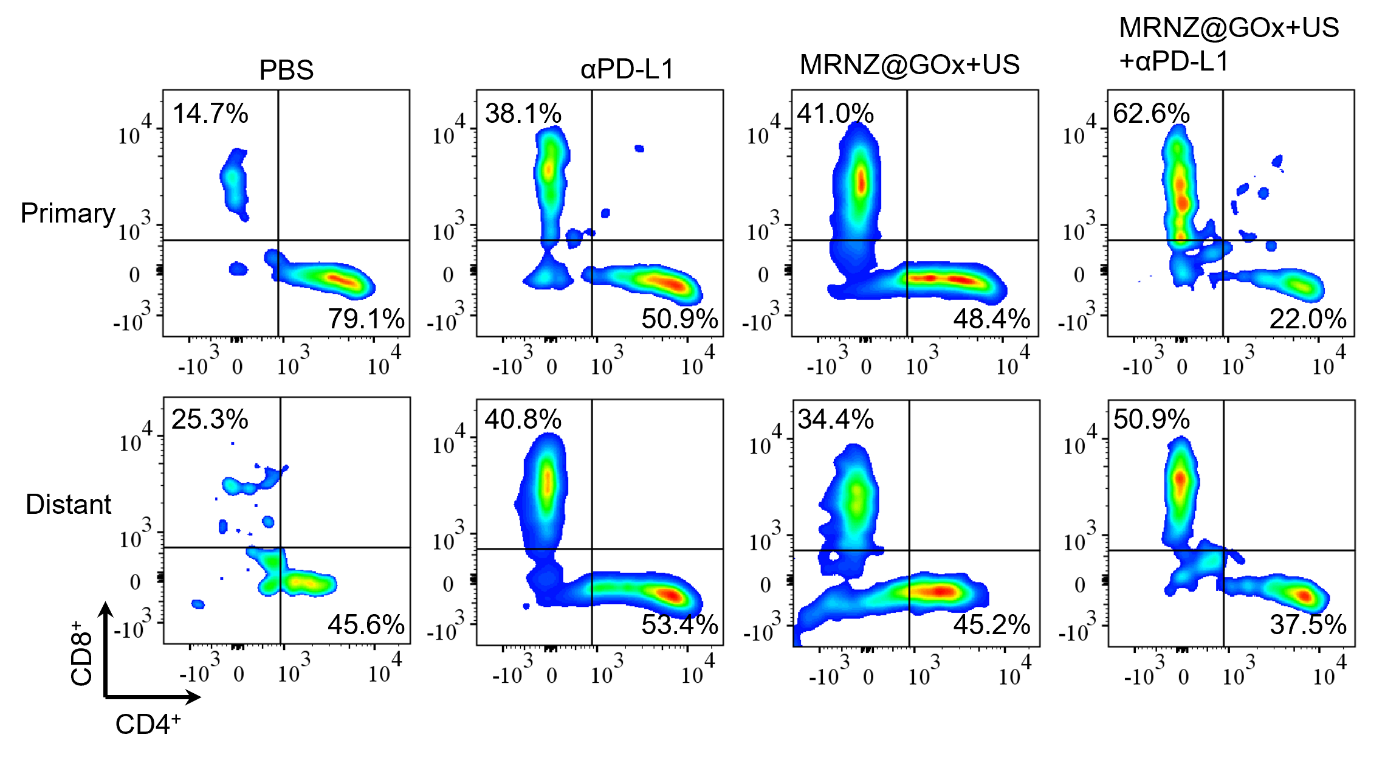


**Figure S23.** Percentage of T cells at bilateral tumor by flow.


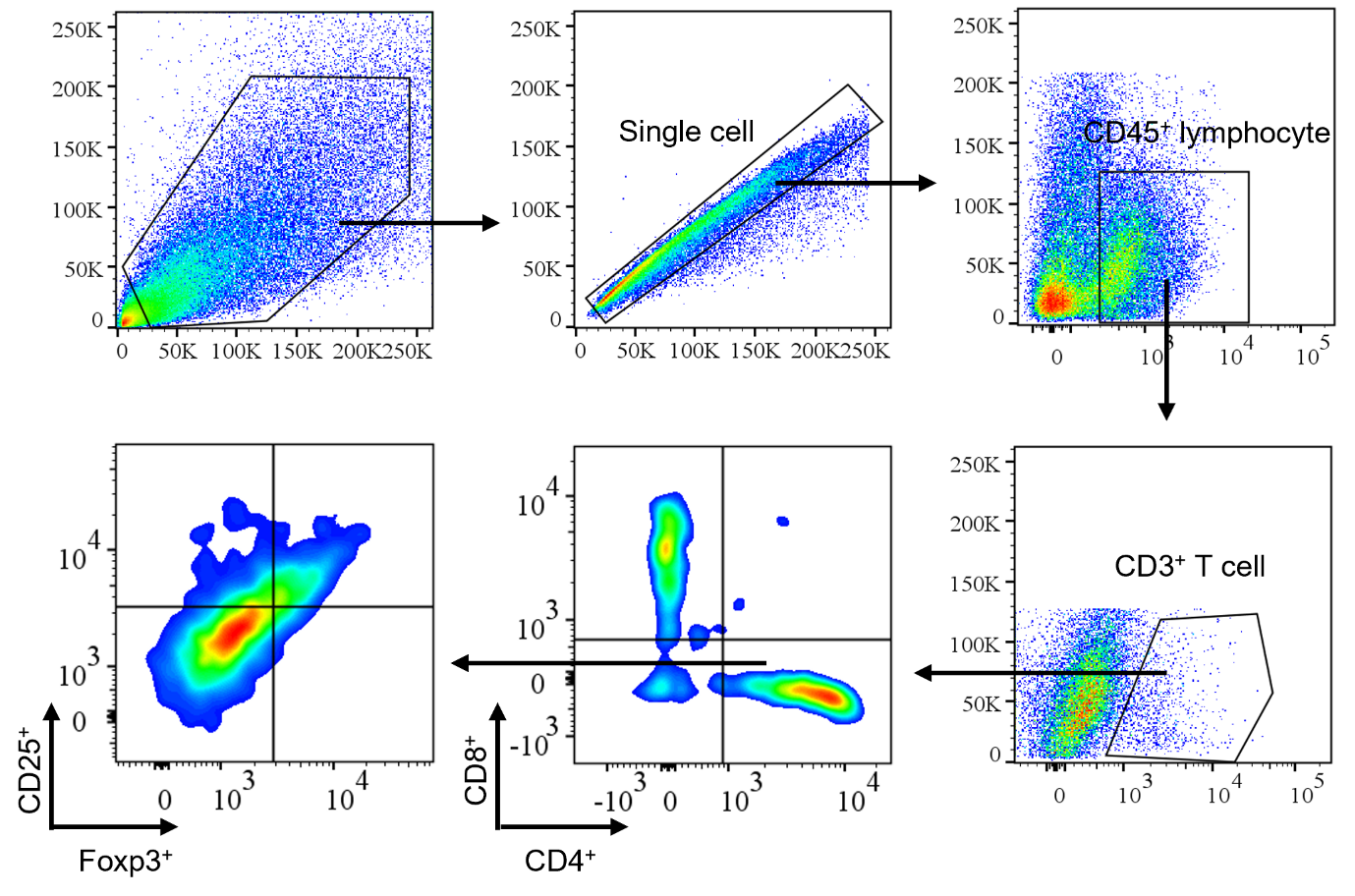


**Figure S24.** (a) Representative flow cytometry gating strategies for T cells and Treg cells.


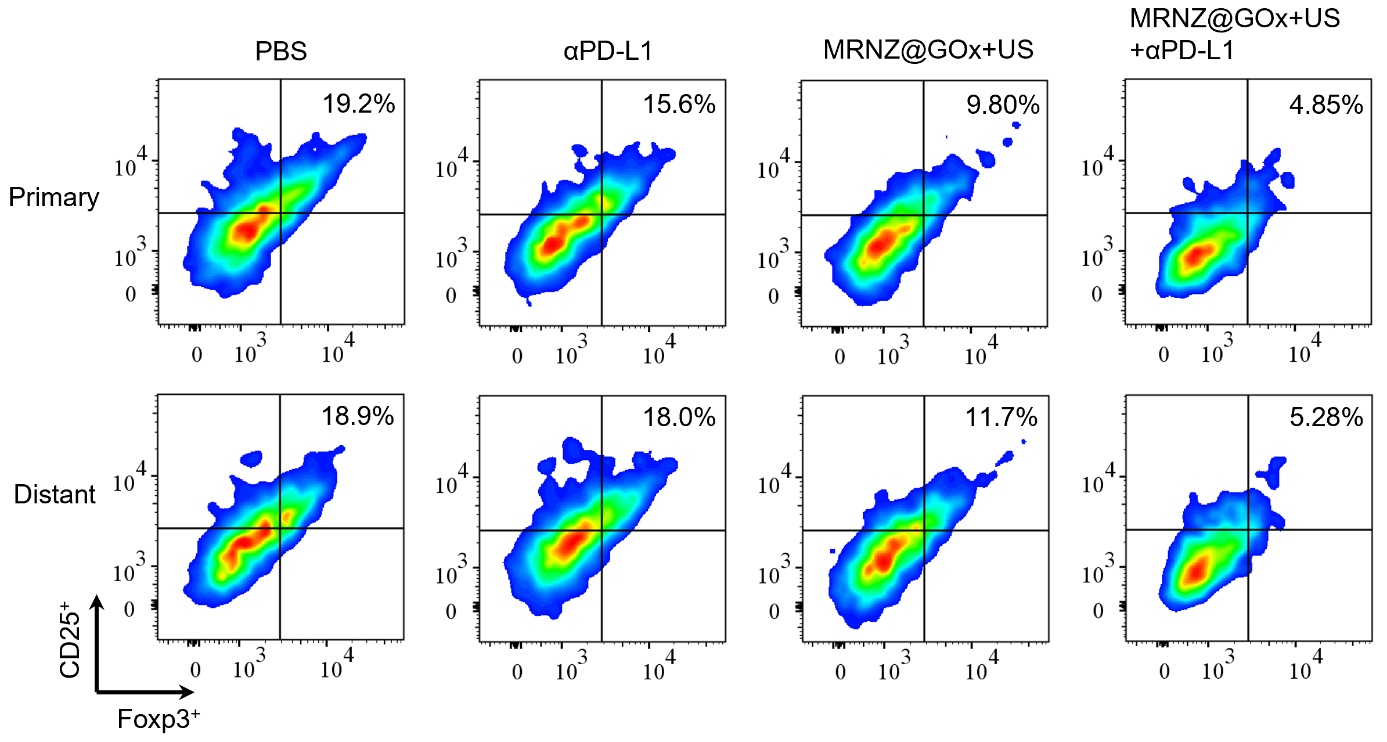


**Figure S25.** Percentage of Treg cells at bilateral tumor by flow.


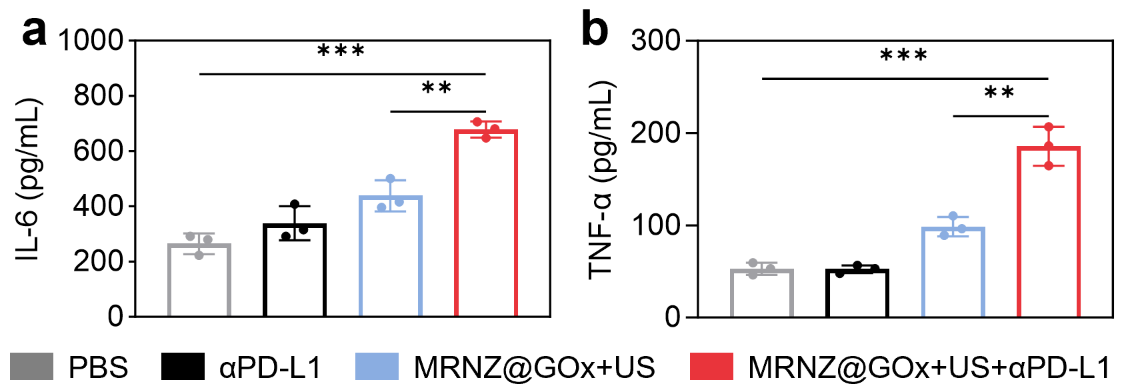


**Figure S26.** The levels of IL-6 (a) and TNF-α (b) from the tumor of mice after different treatment. Data are mean ± SD (n = 3), ***P* < 0.01, ****P* < 0.001 were assessed via one-way ANOVA with Tukey’s multiple comparison tests.


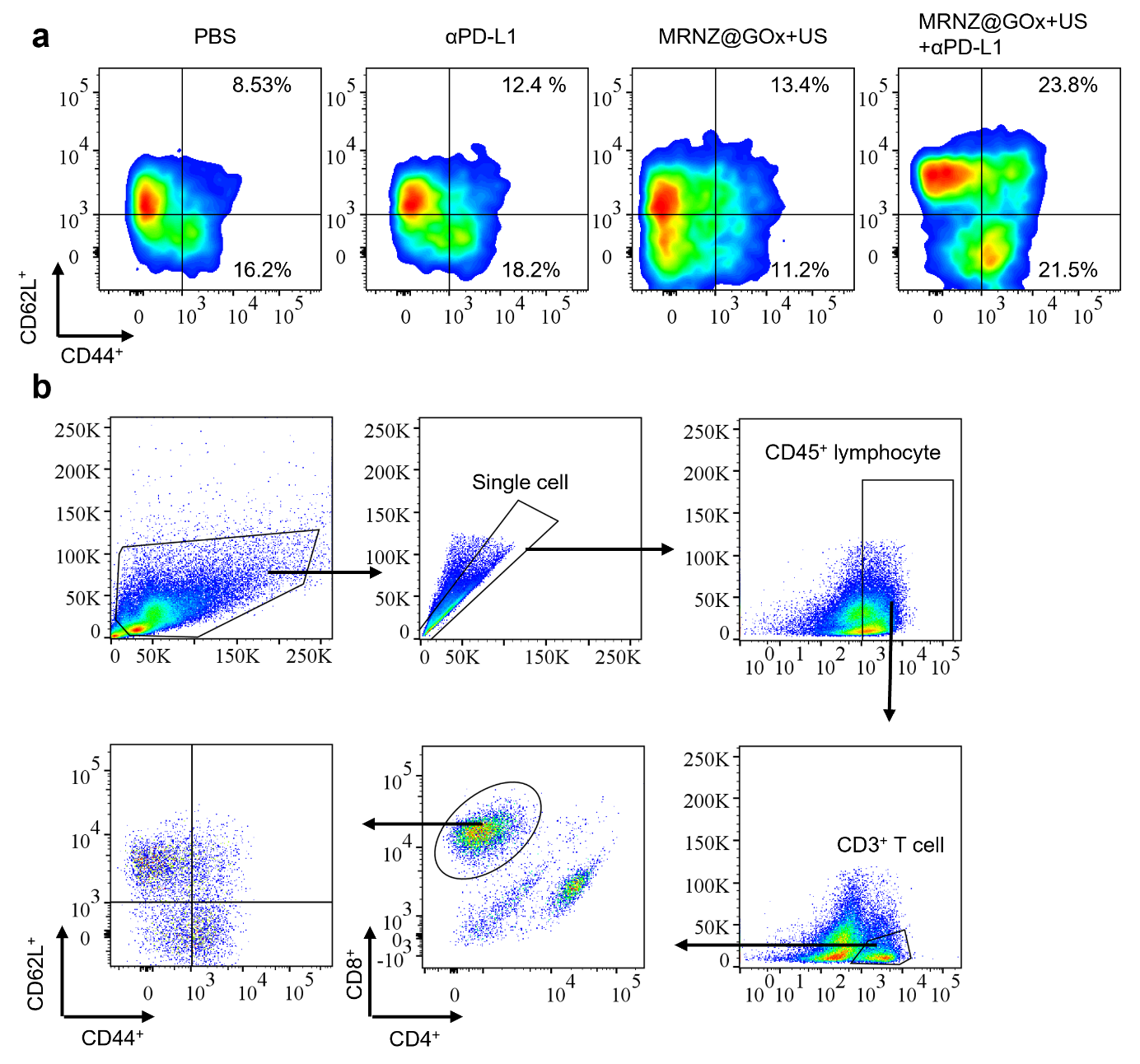


**Figure S27.** (a) Percentage of TCM cells in spleen by flow. (b) Representative flow cytometry gating strategies for TCM cells.


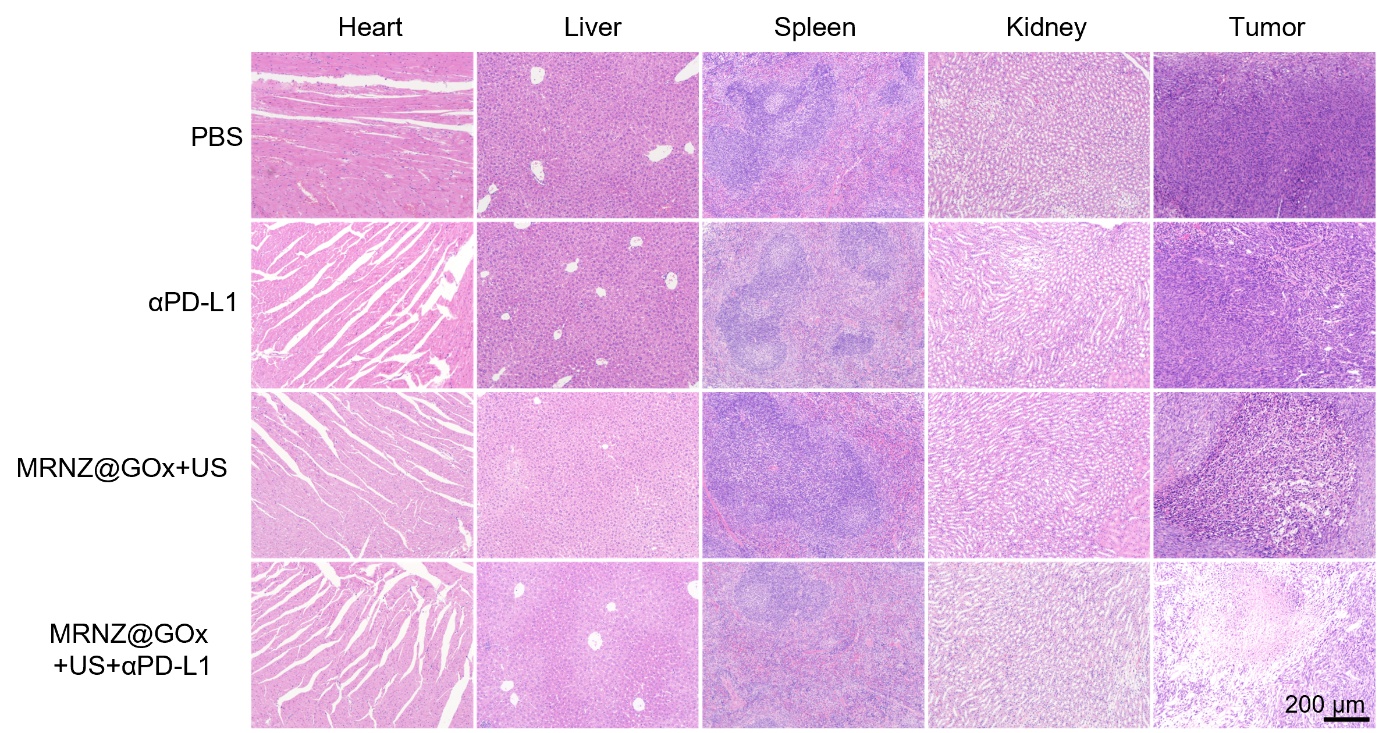


**Figure S28.** H&E staining images of heart, liver, spleen, kidney and tumor of each treatment group at 14 days.

**Table S2**. Michaelis-Menten constant (*K_m_*) and maximum reaction velocity (V_max_) of different nanozymes with POD-like activity

| Materials | *K_m_* (mM) | V_max_ (M·s^-1^) |
| --- | --- | --- |
| This work with US | 36.23 | 3.24 × 10^-7^ |
| This work without US | 97.97 | 1.89 × 10^-7^ |
| Cu_2_O/Au-Pt@MOF@F127^[13]^ | 22.40 | 4.05 × 10^-8^ |
| Zn-SA/CNCl^[14]^ | 147.0 | 5.77 × 10^-6^ |
| Zn-SA/CNCl-HCl^[14]^ | 248.0 | 2.38 × 10^-6^ |
| Zn-SA/CN^[14]^ | 90.00 | 3.25 × 10^-8^ |
| Fe_3_O_4_^[15]^ | 111.0 | 0.15 × 10^-8^ |
| FeN_3_P-SAzyme^[16]^ | 443.0 | 0.20 × 10^-8^ |
| Fe-CDs^[17]^ | 166.0 | 0.49 × 10^-8^ |
| FeNC^[18]^ | 107.0 | 0.32 × 10^-8^ |
| Pdene^[19]^ | 84.40 | 1.26 × 10^-8^ |
| HCFe^[20]^ | 121.6 | 66.86 × 10^-8^ |
| Fe-NC^[21]^ | 0.61 | 10.74 × 10^-6^ |
| Fe-TiO_2_^[22]^ | 1.68 | 2.86 × 10^-4^ |
| Fe_3_O_4_ MNPs^[23]^ | 154 | 5.87 × 10^-6^ |
| Fe@CN^[24]^ | 223.81 | 6.11 × 10^-6^ |
| FOB^[25]^ | 0.17 | 18.11 × 10^-6^ |

**References**

[1] X. de Hatten, Z. Cournia, I. Huc, J. C. Smith, N. Metzler–Nolte, *Chem–Eur. J.* **2007**, 13, 8139.

[2] N. Sieffert, G. Wipff, *Dalton. T.* **2015**, 44, 2623.

[3] R. Krishnan, J. S. Binkley, R. Seeger, J. A. Pople, *J. Chem. Phys*. **1980**, 72, 650.

[4] F. Weigend, R. Ahlrichs, *Phy.s Chem. Chem. Phys.* **2005**, 7, 3297.

[5] S. G. Xu, T. He, J. M. Li, Z. M. Huang, C. W. Hu, *Appl. Catal. B–Environ.* **2021**, 292, 120145.

[6] P. J. Hay, W. R. Wadt, *J. Chem. Phys.* **1985**, 82, 270.

[7] F. Neese, WIREs Comput. *Mol. Sci.* **2025**, 15, e70019.

[8] A. D. Becke, *J. Chem. Phys.* **1993**, 98, 5648.

[9] F. Weigend, R. Ahlrichs, *Phys. Chem. Chem. Phys.* **2005**, 7, 3297.

[10] S. Grimme, S. Ehrlich, L. Goerigk, *J. Comput. Chem.* **2011**, 32, 1456.

[11] S. Grimme, J. Antony, S. Ehrlich, H. Krieg, *J. Chem. Phys.* **2010**, 132, 154104.

[12] J. Ribas–Arino, D. Marx, *Chem. Rev.* **2012**, 112, 5412.

[13] Y. Cheng, Y. D. Xia, Y. Q. Sun, Y. Wang, X. B. Yin, *Adv. Mater.* **2024**, 36, e2308033.

[14] S. Wei, W. Ma, M. Sun, P. Xiang, Z. Tian, L. Mao, L. Gao, Y. Li, *Nat. Commun.* **2024**, 15, 6888.

[15] B. Xu, S. Li, L. Zheng, Y. Liu, A. Han, J. Zhang, Z. Huang, H. Xie, K. Fan, L. Gao, H. Liu, *Adv. Mater.* **2022**, 34, e2107088.

[16] S. Ji, B. Jiang, H. Hao, Y. Chen, J. Dong, Y. Mao, Z. Zhang, R. Gao, W. Chen, R. Zhang, Q. Liang, H. Li, S. Liu, Y. Wang, Q. Zhang, L. Gu, D. Duan, M. Liang, D. Wang, X. Yan, Y. Li, *Nat. Catal.* **2021**, 4, 407-417.

[17] P. Muhammad, S. Hanif, J. Li, A. Guller, F. U. Rehman, M. Ismail, D. Zhang, X. Yan, K. Fan, B. Shi, *Nano Today* **2022**, 45, 101530.

[18] K. Kim, J. Lee, O. K. Park, J. Kim, J. Kim, D. Lee, V. K. Paidi, E. Jung, H. S. Lee, B. Lee, C. W. Lee, W. Ko, K. Lee, Y. Jung, C. Lee, N. Lee, S. Back, S. H. Choi, T. Hyeon, *Adv. Mater.* **2023**, 35, e2207666.

[19] C. Chen, D. Yan, X. Jia, R. Li, L. Hu, X. Li, L. Jiao, C. Zhu, Y. Zhai, X. Lu, *Chem. Sci.* **2024**, 15, 15440-15447.

[20] S. Zhang, X. J. Gao, Y. Ma, K. Song, M. Ge, S. Ma, L. Zhang, Y. Yuan, W. Jiang, Z. Wu, L. Gao, X. Yan, B. Jiang, *Nat. Commun.* **2024**, 15, 10605.

[21] Z. Wang, F. Chen, Z. Wang, M. Wu, M. Jiang, Z. Zheng, Y. Li, Q. Li, Q. Wang, D. Sun, Q. Miao, *Adv. Mater.* **2026**, 38, e18810.

[22] W. Tan, J. Fan, P. Wan, K. Li, *Nano Lett.* **2025**, 25, 13639-13646.

[23] H. Wei, E. Wang, *Anal. Chem.* **2008**, 80, 2250-2254.

[24] W. Wu, H. Zhou, M. Zhang, X. Zhang, M. Huang, S. Tao, W. Du, S. Wang, J. Zhao, X. Zhou, N. Liu, T. Chen, *ACS Nano* **2026**, 20, 8548-8569.

[25] L. Ma, Y. Wang, Y. Chen, D. Xu, R. Han, D. Jiao, H. Xing, D. Wang, X. Yang, *ACS Nano* ***2025***, 19, 28410-28421.
